# The causality between use of glucocorticoids and risk of pancreatitis: a Mendelian randomization study

**DOI:** 10.3389/fimmu.2024.1420840

**Published:** 2024-08-15

**Authors:** Wenfeng Lin, Qiqi Zheng, Xiaorong Wang, Xiaolu Lin, Xixi Ni, Jingye Pan, Maddalena Zippi, Sirio Fiorino, Wandong Hong

**Affiliations:** ^1^ Department of Gastroenterology and Hepatology, The First Affiliated Hospital of Wenzhou Medical University, Wenzhou, Zhejiang, China; ^2^ Department of Infection and Liver Diseases, The First Affiliated Hospital of Wenzhou Medical University, Wenzhou, Zhejiang, China; ^3^ Department of Intensive Care Unit, The First Affiliated Hospital of Wenzhou Medical University, Wenzhou, Zhejiang, China; ^4^ Department of Digestive Endoscopy Center, Fujian Provincial Hospital, Shengli Clinical Medical College of Fujian Medical University, Fuzhou, Fujian, China; ^5^ Unit of Gastroenterology and Digestive Endoscopy, Sandro Pertini Hospital, Rome, Italy; ^6^ Unit of Internal Medicine, Budrio Hospital, Local Health Unit of Bologna, Bologna, Italy

**Keywords:** acute pancreatitis, chronic pancreatitis, glucocorticoid, Mendelian randomization, risk factor

## Abstract

**Background and aim:**

To date, the association between glucocorticoid use and the risk of pancreatitis remains controversial. The aim of this study was the investigation of this possible relationship.

**Methods:**

We carried out a two-sample Mendelian randomization (MR) analysis using GWAS data from European ancestry, East Asian descendants and the FinnGen Biobank Consortium to evaluate this potential causal relationship. Genetic variants associated with glucocorticoid use were selected based on genome-wide significance (p < 5×10-8).

**Results:**

Our MR analysis of European ancestry data revealed no significant causal relationship between glucocorticoid use and AP (IVW: OR=1.084, 95% CI= 0.945-1.242, P=0.249; MR-Egger: OR=1.049, 95% CI= 0.686-1.603, P=0.828; weighted median: OR=1.026, 95% CI= 0.863-1.219, P=0.775) or CP (IVW: OR=1.027, 95% CI= 0.850-1.240, P=0.785; MR-Egger: OR= 1.625, 95% CI= 0.913-2.890, P= 0.111; weighted median: OR= 1.176, 95% CI= 0.909-1.523, P= 0.218). Sensitivity analyses, including MR-Egger and MR-PRESSO, indicated no evidence of pleiotropy or heterogeneity, confirming the robustness of our findings. Multivariable MR analysis adjusted for alcohol consumption, BMI, cholelithiasis and C-reactive protein levels supported these findings. Replicated analysis was performed on datasets from the FinnGen Biobank Consortium and East Asian descendants, and similar results were obtained.

**Conclusions:**

This MR analysis suggests that there is no causal association between glucocorticoid use and the risk of pancreatitis.

## 1 Introduction

Inflammation of the exocrine pancreas, often associated with acute abdominal pain, can lead to multiple organ failure (1, 2). About 80% of cases are classified as mild to moderate with no organ failure after 48 hours, while the remaining 20% progress to severe pancreatitis with a mortality rate of approximately 20% (1, 2). With an increasing global incidence (3), pancreatitis is now the leading cause of hospitalizations related to gastrointestinal disorders worldwide (1). In particular, acute pancreatitis can result from a number of recognized causes, with gallstones and alcohol consumption being the most common. However, the etiology of this condition remains elusive in some cases (2), with a definitive cause being lacking in around 20 per cent of cases (2, 4, 5).

Historically, drug-induced acute pancreatitis has been considered a rare etiology. Recent studies indicate that it may be the third most common cause of the disease, accounting for between 0.1 per cent and 2 per cent of all cases (6, 7). Glucocorticoids (GCs), a widely used group of medications, are prescribed to roughly 2-6% of the population (8). These drugs are known to have a number of negative effects, such as increased diabetes mellitus, osteoporosis and peptic ulcers diseases (9, 10). Furthermore, several case reports have highlighted the onset of pancreatitis in patients receiving glucocorticoid therapy (11–21). Despite this, the pathophysiology and occurrence of glucocorticoid-induced pancreatitis remains poorly investigated and rarely reported. It is difficult to establish a causal relationship between glucocorticoids and pancreatitis (11, 16). Glucocorticoids-induced pancreatitis is remarkably rare, accounting for only 3% of all reported cases of drug-induced pancreatitis according to a literature review (22). This condition is primarily identified by a process of exclusion, and is often considered when there’s a history of glucocorticoid use and after other most common causes of pancreatitis have been ruled out (11, 23). In many of these few reports suggesting glucocorticoids as a potential cause of pancreatitis, the presence of other contributing factors cannot be definitively excluded, making it difficult to attribute the etiology solely to glucocorticoids (24). This difficulty is increased in patients with multiple comorbidities and underlying risk factors, where ruling out more common causes of drug-induced pancreatitis becomes increasingly complex (25). Crucially, some conditions treated with glucocorticoid therapy, such as inflammatory bowel disease (26), systemic lupus erythematosus (27) and Wegener’s granulomatosis (28), may act as risk factors for pancreatitis, leading to confusion in the indication. In addition, the definitive association of pancreatitis with glucocorticoid use is often unconfirmed due to the lack of possibility to repeat tests, especially for ethical reasons (22). A retrospective analysis of patients with systemic lupus erythematosus suggested that glucocorticoids were not responsible for the development of pancreatitis in these cases (29). Conversely, a handful of studies have shown that glucocorticoids may be useful in the prevention and treatment of pancreatitis (24, 30). Nonetheless, the current understanding of glucocorticoid-induced pancreatitis is largely based on theories derived from limited case reports, animal studies and other experimental data (11, 16, 17, 31). The evidence linking glucocorticoids to pancreatitis remains weak, with a significant risk of false-positive results (11, 16, 17, 31). It is therefore essential that large studies are carried out to establish the cause and effect link between the use of glucocorticoids and the risk of pancreatitis.

Mendelian randomization (MR) is a method that uses genetic variation as instrumental variables (IVs) to determine whether an observed association between a risk factor and an outcome is consistent with a causal effect (32). A two-sample MR approach identifies causal effects when exposure and outcome data come from different sources (33). This approach significantly limits residual confounding and is less vulnerable to reverse causation, as genetic variants are inherited at conception. As a result, a trait will typically remain unaffected by other traits (potential confounders or environmental elements). No previous study has investigated the causal relationship between glucocorticoid use and the risk of pancreatitis using MR to our knowledge. Thus, this study attempts to investigate the causal relationship between the use of glucocorticoids and the occurrence of pancreatitis using two-sample MR analysis.

## 2 Methods

### 2.1 Study design and instrument variable selection

Using summary-level data, we conducted a two-sample Mendelian Randomization (MR) study to investigate the causal relationship between glucocorticoid use and pancreatitis employing specific glucocorticoid-related single-nucleotide polymorphisms (SNPs) as instrumental variables (IVs). The main results of the MR analysis in the current study were based on GWAS summary datasets of European ancestry obtained from the study by Sakaue S et al. (34). Replicated analysis was performed on datasets from the FinnGen Biobank Consortium and East Asian descendants.

**Figure 1:**
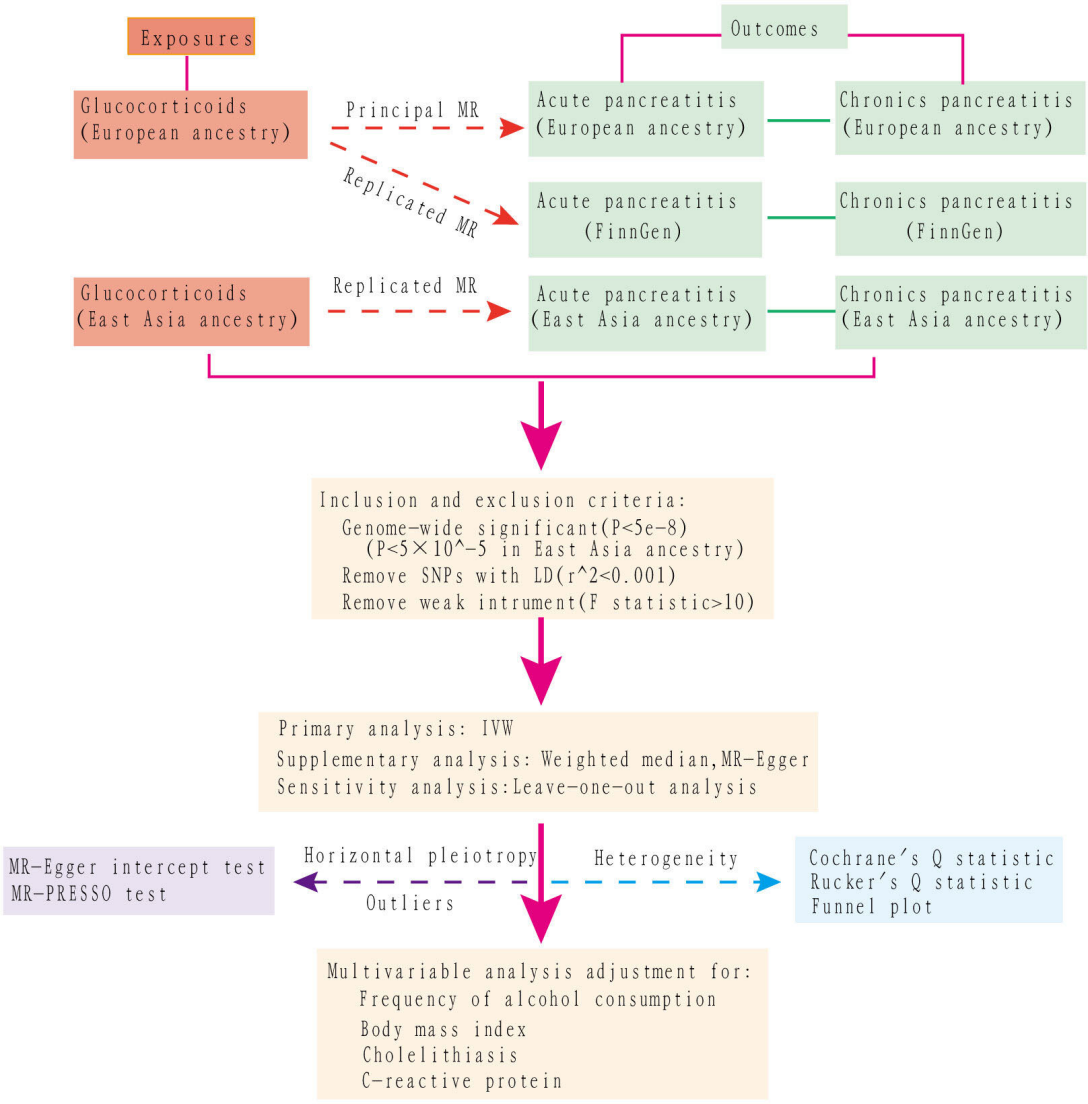
The work flow of this study.

A multivariable MR assessment, adjusting for potential confounders such as preexisting alcohol use, body mass index (BMI), cholelithiasis (gallstones), and C-reactive protein values, was performed to determine the direct causal effect of glucocorticoid use on pancreatitis. The first three factors were identified as etiological contributors to pancreatitis, while the last one assessed the severity of the inflammation. To accurately assess the effects of confounding within the MR framework, the selected IVs must meet three criteria: (I) they should show an association with the exposure variable (the ‘relevance’ assumption); (II) they should not be associated with confounding factors (the ‘independence’ assumption); (III) their influence on the outcome should be mediated solely by the exposure variable, with no additional pathways involved (the ‘exclusion’ restriction). The selection criteria for identifying instrumental variables from SNPs were defined as follows (1): genome-wide significance with P values less than 5×10^-8 was required to ensure the robustness and reliability of these genetic instruments. However, a higher threshold of 5×10^-5 was used for East Asian descendants due to limited qualified data; (2) absence of linkage disequilibrium in SNPs, specified by a default r^2 = 0. 001 within a radius of 10,000 kb, ensuring their independence; and (3) to address potential bias from weak instruments, we calculated the Cragg-Donald F-statistic for each SNP using the formula F-statistic = β^2/SE^2 and excluded SNPs with an F-statistic below 10. In this context, β is the estimate of the exposure effect, while SE is its standard error. The conceptual and analytical flow of this study is illustrated in **Figure 1**.

### 2.2 Data source

**Supplementary Table 1** (34) provides an overview of Genome-Wide Association Studies (GWAS) on various exposures and outcomes. The detailed summary data on glucocorticoid use, acute pancreatitis and chronic pancreatitis in European and East Asian ancestry were extracted from the GWAS conducted by Sakaue S et al. (34). In European ancestry, this study included 17,352 cases of individuals using glucocorticoid (GWAS ID: ebi-a-GCST90019000) with 188,348 controls and analyzed 14,256,400 SNPs. This study also included 3,798 cases of acute pancreatitis (GWAS ID: ebi-a-GCST90018789) and 476,104 controls, analyzing a total of 24,190,697 SNPs. Summary statistics for chronic pancreatitis were extracted from the same GWAS (GWAS ID: ebi-a-GCST90018821). It included 1,424 patients and 476,104 controls, with a total of 24,195,431 SNPs examined. In East Asian descendants, this study included 13,102 cases of glucocorticoid use (GWAS ID: ebi-a-GCST90018780) and 165,624 controls, analyzing 12,454,705 SNPs. This study also included 827 cases of acute pancreatitis (GWAS ID: ebi-a-GCST90018569) and 177,471 controls, evaluating a total of 12,454,648 SNPs. Summary statistics for chronic pancreatitis were extracted from the same GWAS (GWAS ID: ebi-a-GCST90018601), which included 457 patients and 177,471 controls, with a total of 12,454,540 SNPs examined.

The detailed summary level data for acute pancreatitis and chronic pancreatitis were also extracted from the FinnGen Consortium GWAS. For acute pancreatitis, this study included 3,022 patients and 195,144 controls, with a total of 16,380,428 SNPs being investigated (GWAS ID: finn-b-K11_ACUTPANC). Similarly, for chronic pancreatitis, the study included 1,737 patients and 195,144 controls, with 16,380,413 SNPs examined (GWAS ID: finn-b-K11_CHRONPANC).

To clarify direct causal relationships and to reduce potential confounding, genetic instruments for variables such as frequency of alcohol consumption (sample size: 462,346), body mass index (BMI, sample size: 532,396), cholelithiasis (gallstones, sample size: 404,405) and C-reactive protein levels (sample size: 353,466) were acquired from the most comprehensive and recent studies (34–39). The first three variables above serve as etiological contributors to pancreatitis, with the last variable indicating the severity of the inflammatory response.

Detailed data sources for glucocorticoid administration, acute and chronic pancreatitis, frequency of alcohol consumption, BMI, cholelithiasis and C-reactive protein are meticulously documented in **Supplementary Table 1**.

### 2.3 Statistical analysis

The primary analytical approach applied in this study was the Inverse Variance Weighted (IVW) method, which assesses the effect of SNPs associated with glucocorticoid use on pancreatitis risk by aggregating individual Wald ratios to achieve unbiased causality in the absence of horizontal pleiotropy (40). Supplementary analyses using the weighted median and MR-Egger methods have also been performed to corroborate these findings (41, 42).

The influence of horizontal pleiotropy on risk estimation and the identification of potential confounders was evaluated by means of the MR-Egger intercept test (41). Heterogeneity of results was assessed using Cochrane’s Q statistic for IVW analysis and Rucker’s Q statistic for MR-Egger analysis (43). In addition, we performed a leave-one (SNP)-out analysis to identify and exclude outliers, potentially biasing a causal relationship and we systematically omitted each SNP and recalculated effect sizes, using the IVW method. Funnel plots were generated to visually assess the heterogeneity of the results, with a symmetric distribution around the vertical axis. This type of configuration indicated the absence of bias. The Mendelian randomization pleiotropy residual sum and outlier (MR-PRESSO) test was also used to identify horizontal pleiotropic outlier SNPs, providing identical results to IVW after outlier removal (44). To minimize the impact of horizontal pleiotropy on the results, each individual SNP was examined individually in the LDtrait human genotype-phenotype databases (45). This process allowed us to identify and exclude risk factors shared with glucocorticoid use, such as serum triglyceride levels (46), cholangitis (46) and alcohol consumption (46).

Multivariable MR analysis can be used to investigate the causality of multiple exposures imposed by a genetic tool on the same outcome variable. In clinical practice, alcohol and cholelithiasis are known to be common etiologies of pancreatitis, while BMI and CRP are risk and predictive factors of disease severity in patients with pancreatitis, respectively (46–49). These indexes may act as possible confounding factors that bias the results of the MR analysis. Therefore, we performed multivariable MR analysis to remove potential confounding bias. All Mendelian randomization analyses were performed using the TwoSampleMR package in R version 4.1.2, with P values less than 0.05 considered statistically significant.

## 3 Results

### 3.1 MR analysis of GWAS summary datasets of European ancestry

#### 3.1.1 Causal association of glucocorticoid usage with AP

In this analysis, we employed 27 SNPs as instrumental variables to assess the impact of glucocorticoid use through MR analysis. Each SNP had an F-statistic greater than 10, exceeding the threshold for a ‘weak instrumental variable’ (F-statistic value less than 10), thereby mitigating concerns about weak instrument bias in our results.

**Figure 2:**
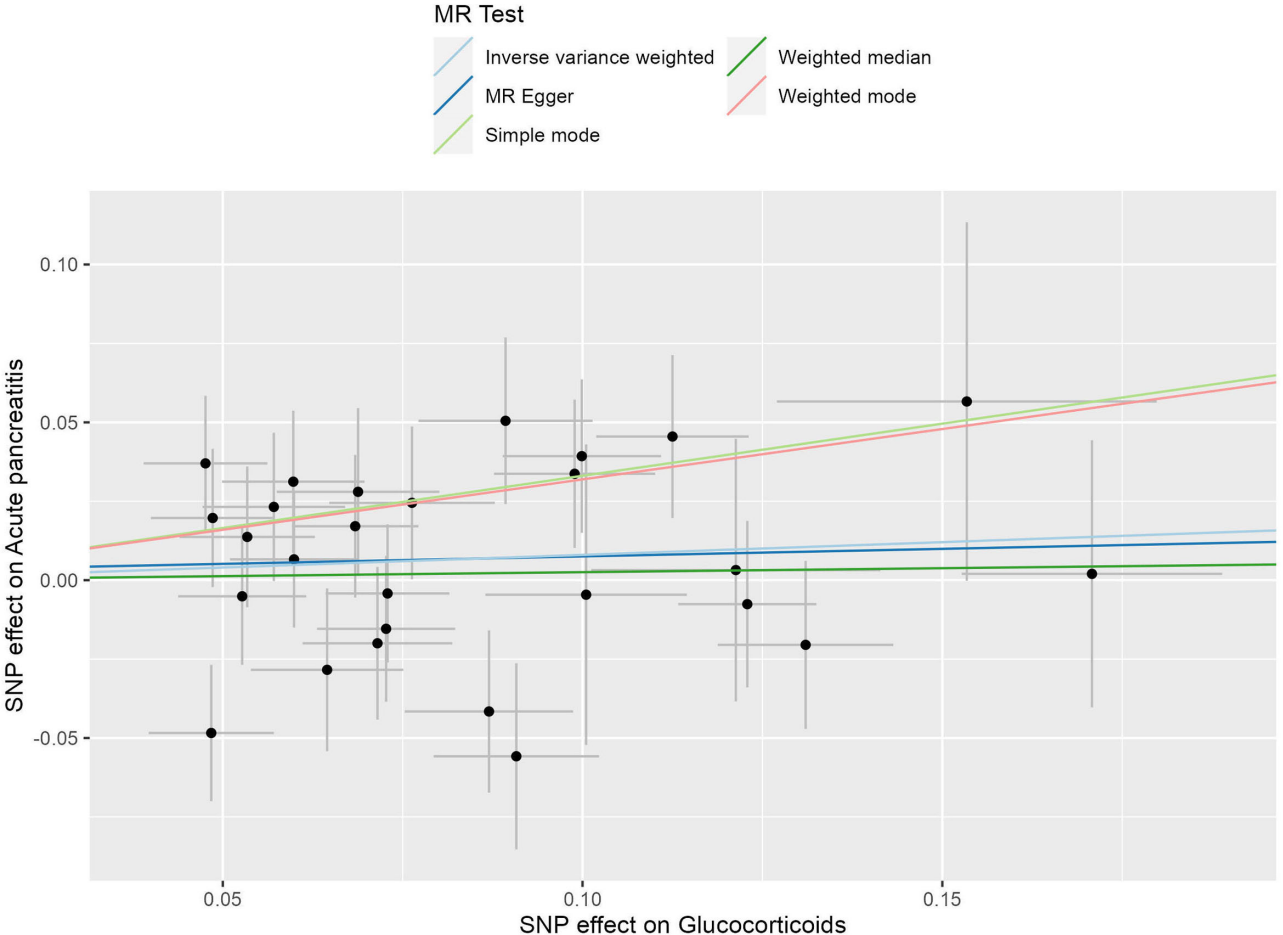
The scatter plot illustrates the causal effect of glucocorticoid usage on the risk of acute pancreatitis (AP) using GWAS summary data sets of European ancestry. The slope of the line indicates the strength of this causal relationship. MR denotes Mendelian randomization.

**Figure 3:**
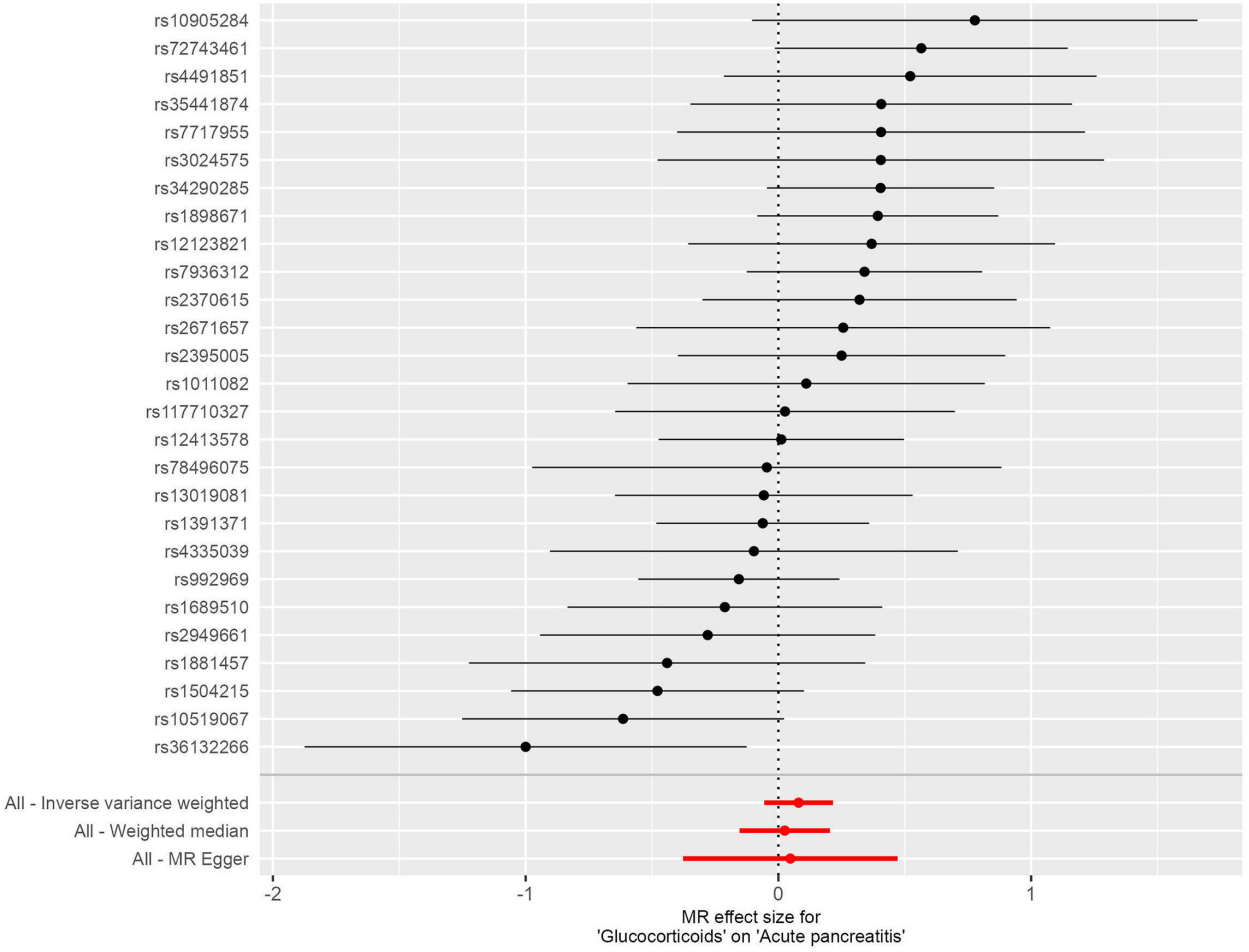
Forest plots illustrating the causal relationship between individual SNPs and the risk of acute pancreatitis (AP) using GWAS summary data sets of European ancestry.

There was no statistically significant causal relationship between glucocorticoid use and the development of acute pancreatitis (AP) using the inverse variance weighted (IVW) method (odds ratio (OR) = 1.084, 95% confidence interval (CI) = 0.945-1.242, P = 0.249), as depicted in **Figure 2**. Similarly, MR-Egger regression analysis (OR = 1.049, 95% CI = 0.686-1.603, P = 0.828) and the weighted median method (OR=1.026, 95% CI= 0.863-1.219, P=0.775) supported these findings, as presented in **Figure 3**. No horizontal pleiotropic outlier SNPs were identified by MR-PRESSO Global test (P*_Global test_* =0.123).

#### 3.1.2 Causal association of glucocorticoid usage with CP

**Figure 4:**
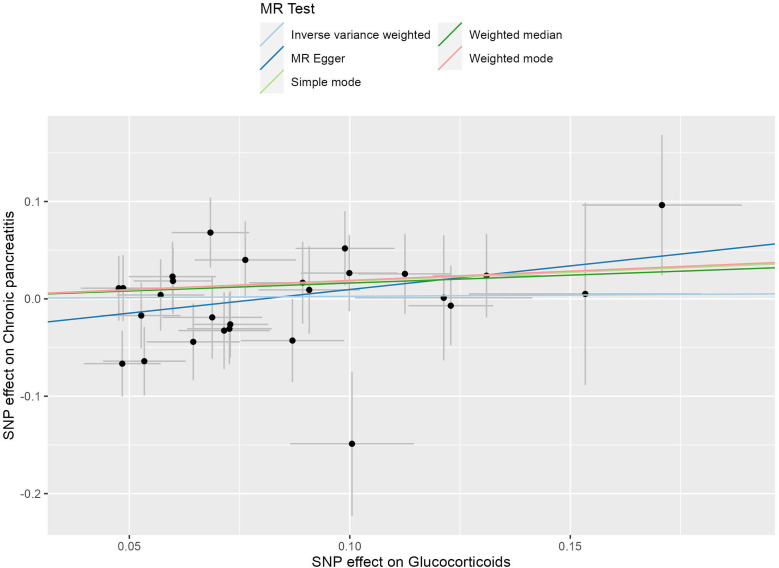
The scatter plot illustrates the causal effect of glucocorticoid usage on the risk of chronic pancreatitis (CP) using GWAS summary data sets of European ancestry. The slope of the line indicates the strength of this causal relationship. MR denotes Mendelian randomization.

**Figure 5:**
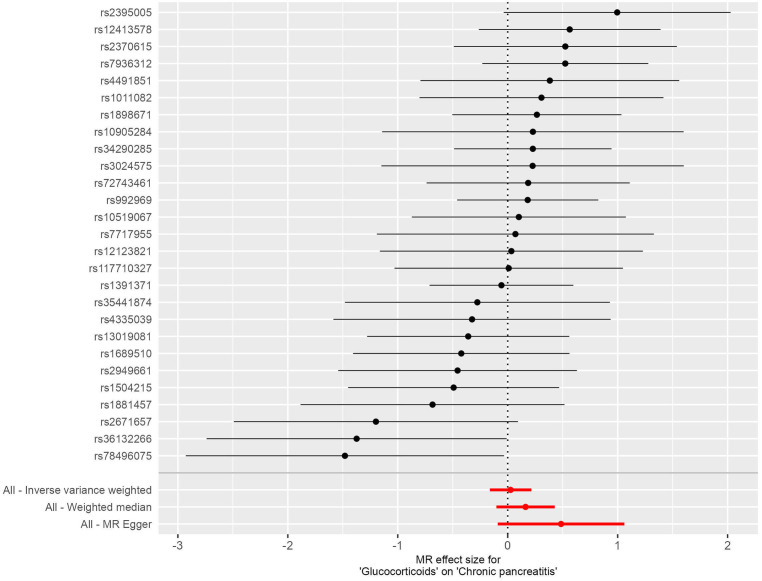
Forest plots illustrating the causal relationship between individual SNPs and the risk of chronic pancreatitis (CP) using GWAS summary data sets of European ancestry.

In this investigation, we included 26 SNPs as instrumental variables to assess the effect of glucocorticoid use in an MR analysis. All SNPs had F-statistics greater than 10, exceeding the threshold for weak instrumental variables. Therefore, concerns about weak instrumental bias in our results are considered negligible. The IVW method, as shown in **Figure 4**, did not reveal a substantial causal relationship between glucocorticoid use and the incidence of CP, with an OR of 1.027 and a 95% CI ranging from 0.850 to 1.240, resulting in a P value of 0.785. Similarly, both the MR-Egger regression yielded an OR of 1.625 (95% CI: 0.913-2.890; P=0.111) and the weighted median approach indicated an OR of 1.176 (95% CI: 0.909-1.523; P=0.218), supporting these findings (**Figure 5**). No horizontal pleiotropic outlier SNPs were identified by MR-PRESSO Global test (P*_Global test_* = 0.493).

#### 3.1.3 Heterogeneity and sensitivity analysis

Cochran’s Q statistics indicated the absence of significant heterogeneity in our results, as all P values exceeded 0.05 (**Supplementary Table 2**). In addition, the symmetric funnel plots generated for individuals with AP and CP further confirmed the lack of heterogeneity in our results (**Supplementary Figures 1**, **2**). To assess potential pleiotropy, we used the MR Egger intercept test, which yielded intercepts that were not statistically different from zero (all p-values > 0.05; p=0.874 for AP and p=0.243 for CP), suggesting no evidence of horizontal pleiotropy in our investigation. Furthermore, the leave-one-out analysis showed that no single SNP significantly influenced the overall causal estimate (**Supplementary Figures 3**, **4**). Detailed information on the MR analyses can be found in **Supplementary Table 2**.

#### 3.1.4 Multivariable MR analysis

**Figure 6:**
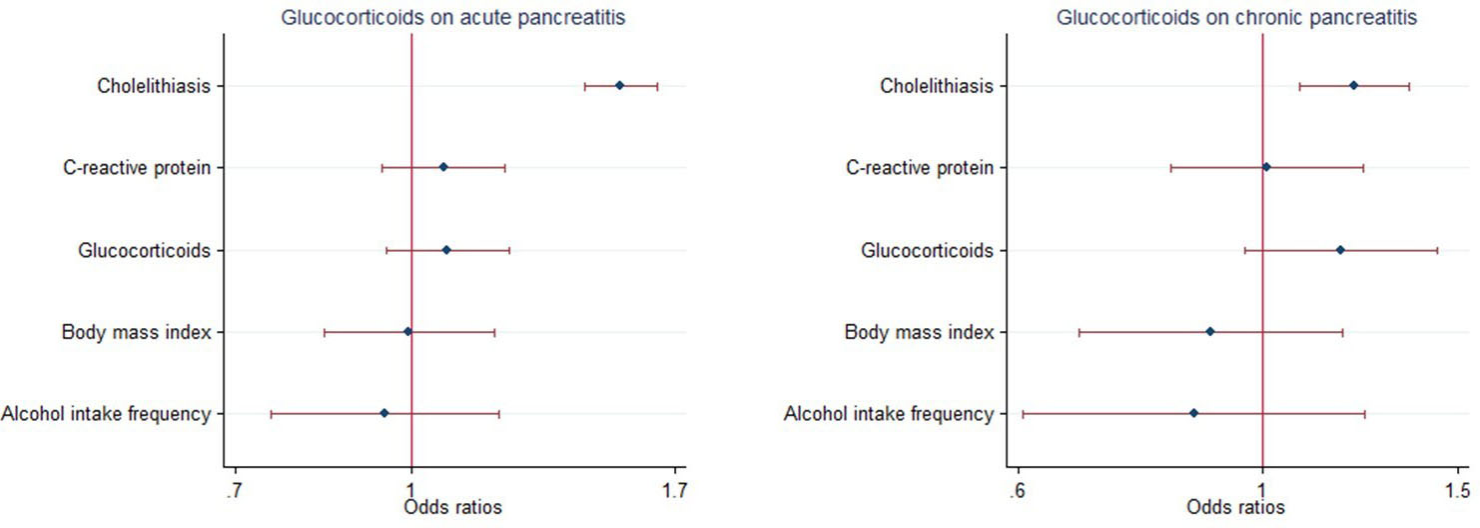
Multivariable Mendelian randomization of glucocorticoid usage on the risk of pancreatitis using GWAS summary data sets of European ancestry. Error bars represent 95% confidence intervals. AP, acute pancreatitis; CP, chronic pancreatitis.

Furthermore, following adjustment for frequency of alcohol consumption, body mass index (BMI), cholelithiasis (gallstones) and C-reactive protein levels, multivariable MR analysis showed that there was no direct effects of glucocorticoid use either on the risk of AP (OR = 1.074, 95% CI = 0.948-1.216, P = 0.263, **Figure 6**; **Supplementary Table 3**) or risk of CP (OR =1.176, 95% CI =0.962-1.438, P =0.114; **Figure 6**, **Supplementary Table 3**).

### 3.2 Replicated MR analysis of GWAS summary datasets from the FinnGen Biobank Consortium and East Asian descendants

#### 3.2.1 Causal association of glucocorticoid use in relation to AP

Using the same instrumental variables of European ancestry, there was no statistically significant causal relationship between glucocorticoid administration and development of acute pancreatitis (AP) using the inverse variance weighted (IVW) method (OR = 1.130, 95% CI = 0.921-1.386, P = 0.243), as shown in **Supplementary Figure 5** in the FinnGen Biobank Consortium. Similarly, the MR-Egger regression analysis (OR = 1.094, 95% CI = 0.567-2.110, P = 0.791) and the weighted median method (OR = 0.963, 95% CI = 0.771-1.202, P = 0.737) supported these findings, as shown in **Supplementary Figure 6**. The MR-PRESSO method identified one outlier SNP (rs10905284, P*_Global test <_* 0.001). However, outlier correction shows similar OR estimates to the IVW method after removal of this outlying SNP (OR = 1.088, 95% CI = 0.917-1.2191, P = 0.343).

When employing the instrumental variables of the East Asian descendants, there was no statistically significant causal relationship between glucocorticoid administration and the development of acute pancreatitis (AP), by means of the inverse variance weighted (IVW) method (OR = 0.859, 95% CI = 0.682-1.083, P = 0.199), as shown in **Supplementary Figure 7** in East Asian descendants. Similarly, MR-Egger regression analysis (OR = 0.796, 95% CI = 0.357-1.775, P = 0.580) and the weighted median method (OR = 0.962, 95% CI = 0.698-1.325, P = 0.812) supported these findings, as illustrated in **Supplementary Figure 8**. No horizontal pleiotropic outlier SNPs were identified by the MR-PRESSO Global test ((P*_Global test_* = 0.602).

#### 3.2.2 Causal association of glucocorticoid usage with CP

Using the same instrumental variables of European ancestry, the IVW method, as shown in **Supplementary Figure 9**, indicated no substantial causal relationship between glucocorticoid use and incidence of CP, with an OR of 0.982 and a 95% CI ranging from 0.798 to 1.209 and with a P value of 0.864 in the FinnGen Biobank Consortium. Similarly, both the MR-Egger regression showed an OR of 1.429 (95% CI: 0.748-2.730; P=0.291) and the weighted median approach showed an OR of 0.996 (95% CI: 0.750-1.324; P=0.979), supporting these findings (**Supplementary Figure 10**). No horizontal pleiotropic outlier SNPs were identified by the MR-PRESSO Global test (P*_Global test_* = 0.269).

The evaluation of the instrumental variables in East Asian descendants produced no statistically significant causal relationship between glucocorticoid administration and the development of CP, using the inverse variance weighted (IVW) method (OR = 1.038, 95% CI = 0.761-1.415, P = 0.816), as shown in **Supplementary Figure 11** in East Asian descendants. Similarly, MR-Egger regression analysis (OR = 0.660, 95% CI = 0.223-1.930, P = 0.452) and the weighted median method (OR = 1.064, 95% CI = 0.674-1.679, P = 0.791) supported these findings, as illustrated in **Supplementary Figure 12**. No horizontal pleiotropic outlier SNPs were identified by the MR-PRESSO Global test (P*_Global test_* = 0.602).

#### 3.2.3 Heterogeneity and sensitivity analysis

Significant heterogeneity was identified by Cochran’s Q statistic for AP in the FinnGen Biobank Consortium (P_Q.Egger_=0.0012; P_Q.IVW_=0.0019). Cochran’s Q statistic indicated the absence of significant heterogeneity in our other results, as all P values exceeded 0.05. In addition, the symmetric funnel plots generated for individuals with AP and CP further confirmed the lack of heterogeneity in our results except for AP in the FinnGen Biobank Consortium (**Supplementary Figures 13**, **14**). To assess potential pleiotropy, we used the MR Egger intercept test, which yielded intercepts that were not statistically different from zero (all p-values > 0.05), suggesting no evidence of horizontal pleiotropy in our investigation. Furthermore, the leave-one-out analysis showed that no single SNP significantly influenced the overall causal estimate (**Supplementary Figures 15**, **16**). Detailed information on the MR analyses can be found in **Supplementary Table 4**.

In East Asian descendants, Cochran’s Q statistic also indicated no significant heterogeneity, with all P values above 0.05 (**Supplementary Table 5**). Symmetric funnel plots for individuals with AP and CP further supported the absence of heterogeneity (**Supplementary Figures 17**, **18**). The MR Egger intercept test yielded intercepts that were not statistically different from zero (all P values > 0.05; P=0.845 for AP and P=0.392 for CP), indicating no horizontal pleiotropy. The leave-one-out analysis showed that no single SNP significantly influenced the overall causal estimate (**Supplementary Figures 19**, **20**). Detailed information on the MR analyses can be found in **Supplementary Table 5**.

#### 3.2.4 Multivariable MR analysis

Furthermore, after adjustment for frequency of alcohol consumption, body mass index (BMI), cholelithiasis (gallstones) and C-reactive protein levels, multivariable MR analysis showed no direct effect of glucocorticoid use on the risk of either AP (OR = 1. 065, 95% CI = 0.911-1.244, P = 0.429, **Supplementary Figures 21**, **Supplementary Table 6**) or the risk of CP (OR =1.090, 95% CI =0.885-1.343, P =0.418; **Figure 6**; **Supplementary Table 6**) in the FinnGen Biobank Consortium. Multivariable MR analysis was not performed in East Asian offspring due to limited qualifying data.

## 4 Discussion

Establishing a diagnosis for drug-induced pancreatitis poses significant diagnostic challenges. This pathological condition is quite rare and it may present with different clinical course and severity.

Therefore, it is often difficult or ethically unjustifiable to use rechallenge to test for a causal relationship between a potentially dangerous drug and the development of pancreatitis, mainly in its acute form (1, 2, 50, 51). The hypothesis that glucocorticoids contribute to or correlate with pancreatitis in humans has been emphasized by several Authors for many years (11–21). However, our current understanding of glucocorticoid-induced pancreatitis primarily relies on limited individual case series, animal research and other experimental findings (11, 16, 17, 24, 31). Consequently, studies assessing the possible association between glucocorticoids and pancreatitis provide no definitive conclusions, due to the risk of potential false positive results (11, 16, 17, 24, 31). Furthermore, few robust and large-scale studies investigating glucocorticoid-induced pancreatitis are available to date. Therefore, this circumstance makes the specific mechanisms associated with this condition largely unexplored and understood (13, 52). Among the few large-scale investigations to date, a population-based nested case-control study (13) examined 6,161 cases of acute pancreatitis along with 61,637 controls to explore the relationship between oral glucocorticoid use and incidence rates of acute pancreatitis. This study showed an increased probability of acute pancreatitis development in individuals currently using oral glucocorticoids compared to non-users (OR 1.53; 95% CI 1.27-1.84), suggesting that taking these drugs increases the risk of this disease. Nonetheless, the investigators also underlined that their study was subject to limitations, including the potential misestimation of prescribed medication use and the inability to adjust for confounding variables. The association with the use of glucocorticoids and the incidence of acute pancreatitis was investigated in another large study using the US Food and Drug Administration Adverse Event Reporting System (FAERS) (14). In this study, 8,437,343 cases were analyzed and 44,893 cases of acute pancreatitis were identified in patients who were taking various medications, including glucocorticoids. A pharmacological and epidemiological approach was used in this study. It concluded that glucocorticoid treatment was associated with an increased risk of having acute pancreatitis. This circumstance introduces some limitations to this study, such as susceptibility to underreporting, selective reporting bias and an inability to adjust for all confounding factors, thereby precluding definitive conclusions on causal relationship between glucocorticoids and acute pancreatitis. Furthermore, individuals suffering from this disease while on this medication frequently exhibit prominent predisposing factors for the development of this pathological condition, such as alcohol abuse, systemic vasculitis, due to immunological responses, and concurrent use of pharmacological substances recognized to induce pancreatitis, in addition to the drug under suspicion (22, 24, 31). It is also suggested that glucocorticoids may be involved in the onset of acute pancreatitis in people receiving this type of therapy to treat autoimmune diseases such as systemic lupus erythematosus (SLE) (53). However, it is worth noting that a significant proportion (approximately 8%) of SLE patients experience acute pancreatitis regardless of whether they have received glucocorticoids (53). Likewise, the development of acute pancreatitis in cancer patients has been linked to the use of glucocorticoids given as an anti-emetic during chemotherapy (54). However, it should be noted that these patients are often taking antineoplastic medications at the same time. These drugs are known to independently cause acute pancreatitis (54).

The pathophysiological mechanisms underlying glucocorticoid-induced pancreatitis remain poorly understood, although several theories have been proposed to elucidate its etiology (24). Some studies suggest that alterations in calcium metabolism within pancreatic cells may contribute to the development of this condition (19, 21), while others hypothesize that glucocorticoids promote the production of viscous protein-rich secretions, leading to blockage of pancreatic ductules and subsequent localized inflammation (55). Additional evidence indicates that intravenous administration of ACTH, hydrocortisone, or prednisolone can decrease pancreatic volume as well as bicarbonate and amylase secretion (56). It has also been postulated that glucocorticoids may increase total lipid levels, potentially triggering acute pancreatitis (57–60). However, these hypotheses are primarily based on individual animal experiments or clinical observations; several studies have produced conflicting or negative results (61–69). For example, high-dose methylprednisolone has been shown to reduce pancreatic inflammation and edema in animal models by inhibiting cytokine release and leukocyte activation (70). Dexamethasone has been shown to protect pancreatic tissue through its anti-inflammatory effects and inhibition of several inflammatory mediators (71). These inconsistencies cast doubt on the validity and strength of these clinical and laboratory deductions.

Furthermore, emerging research suggests that glucocorticoids exhibit therapeutic potential in the management of pancreatitis, particularly during its early phase. This step is characterized by the development of a significant phlogosis (72), a process which may trigger systemic inflammatory responses and impairs organ functionality (73). As potent anti-inflammatory agents, glucocorticoids have demonstrated efficacy across several inflammatory conditions (74–76). Notably, in animal models of AP, glucocorticoid treatment has shown promising therapeutic outcomes by improving survival rates (71, 77–81), although the underlying mechanisms remain unclear. The speculated pathophysiological pathways through which glucocorticoids may exert their effects in treating pancreatitis include suppression of inflammatory mediators (82), attenuation of endotoxin-induced damage (83), enhancement of microcirculation (84), scavenging oxygen free radicals (85), reduction of nitric oxide levels (86) and NF-kappa B activities (87, 88), as well as induction of acinar cell apoptosis (89–91). These insights underscore the potential role of glucocorticoids in improving outcomes associated with pancreatitis. For decades, there has been extensive research into the use of glucocorticoids in the treatment of AP (92), particularly this medication is considered a conventional treatment in autoimmune pancreatitis (93). Based on the best we know, Stephenson et al. were the first to report the therapeutic benefits of glucocorticoids in human hemorrhagic acute pancreatitis (AP) in 1952 (94). Subsequently, numerous corroborating clinical trials and case reports published in the literature. For example, one study demonstrated how combining dexamethasone with a traditional Chinese herbal concoction reduced the risk of acute respiratory distress syndrome (ARDS) in SAP (severe acute pancreatitis) patients (95), while a meta-analysis of six Chinese trials suggested that corticosteroids could improve patient outcomes in SAP cases (96). The aforementioned studies collectively suggest that glucocorticoids may confer therapeutic benefits in the management of pancreatitis, thereby raising questions about the causal relationship between glucocorticoid use and the potential initiation of pancreatitis.

It is crucial to acknowledge that the majority of existing research primarily includes observational studies. Besides the aforementioned studies (13, 97), several smaller observational studies have discussed the effect of glucocorticoids on pancreatitis. For example, Iqbal et al. (98) reported a case of pancreatitis induced by high-dose glucocorticoids in a patient being treated for optic neuritis. This case highlighted the need for vigilance on the part of doctors, but its applicability was limited by the fact that it was a single case. Similarly, Ataallah et al. (17) documented a case of acute pancreatitis in a patient with idiopathic immune purpura who had recently been treated with steroids. This report highlights the diagnostic challenges in such patients, but being a single case study, its wider implications are limited. Observational studies are inherently susceptible to biases such as confounding, selection, recall, measurement and reporting bias, and temporal issues (99–101). Considering the inherent limitations of observational studies in establishing causation or fully accounting for confounding factors, caution must be taken into account when these findings are interpreted. To address this limitation and establish a causal link between glucocorticoid use and pancreatitis risk, we conducted a MR study. Unlike traditional observational studies, this method minimizes bias and reduces the risk of reverse causality by using genetic variants as instrumental variables (41, 102–104). This approach provides stronger evidence of causality and allows for a more robust assessment of the long-term effects of glucocorticoid use (41, 102–104). This methodological rigor increases the reliability of our findings and provides clearer insights into the true impact of glucocorticoid use on the risk of pancreatitis (41, 102–104). Our investigation found no significant evidence of a causal association between glucocorticoid use and the risk of acute pancreatitis (AP) and chronic pancreatitis (CP), as determined by rigorous statistical methods including inverse variance weighted (IVW), MR Egger regression, weighted median approach and MR-PRESSO. Across the GWAS pooled datasets from European ancestry, the FinnGen Biobank Consortium and East Asian descendants, Cochran’s Q statistics indicated no significant heterogeneity in most outcomes (all P values > 0.05), except for AP in the FinnGen Biobank Consortium (PQ.Egger=0.0012; PQ.IVW=0.0019). Symmetric funnel plots for individuals with AP and CP further confirmed the lack of heterogeneity in these populations (**Supplementary Figures 1**, **2**, **13**, **14**, **17**, **18**). To assess potential pleiotropy, we used the MR Egger intercept test, which yielded intercepts that were not statistically different from zero (all P values > 0.05), suggesting no evidence of horizontal pleiotropy. In addition, MR-PRESSO identified one outlier SNP (rs10905284, PGlobal test < 0.001), but showed similar OR estimates to the IVW method after removing this outlier (OR = 1.088, 95% CI = 0.917-1.2191, P = 0.343), reinforcing the robustness of our findings. The leave-one-out analysis further demonstrated that no single SNP significantly influenced the overall causal estimate across all datasets (**Supplementary Figures 3**, **4**, **15**, **16**, **19**, **20**). Detailed information on the MR analyses can be found in **Supplementary Tables 2**, **4** and **5**. Overall, these results demonstrate the robustness of our findings, which are consistent across different populations and methodologies. The inclusion of the MR-PRESSO results further validates our findings by addressing potential pleiotropy and confirming the stability of our estimates after outlier correction. Our findings differ from observational studies suggesting an increased risk of pancreatitis with glucocorticoid use. These discrepancies may be due to methodological differences, residual confounding, or limitations of observational data.

Our MR study used genetic instruments to investigate the causal relationship between glucocorticoid administration and the risk of pancreatitis. As far as we know, this is the first reported study to apply the MR method and visual representations in order to explore the causality effects of glucocorticoid usage on pancreatitis risk. The primary strength of our investigation lies in its employment of MR analytical approach, which effectively mitigates confounding biases inherent in retrospective studies and provides more compelling evidence. Unlike traditional observational studies, MR analysis significantly reduces the possibility of reverse causation (41, 102–104). However, it is important to recognize some limitations within our study. Our study does not take into account variations in glucocorticoid dosage, duration of use, or treatment regimens for different conditions. Future studies should take these factors into account to provide a more complete understanding of the relationship between glucocorticoid use and the risk of pancreatitis. Moreover, our findings are based on summary level data and should be interpreted with caution, given the assumptions about genetic tools and potential biases inherent in MR analyses. Although the MR-Egger intercept test showed no evidence of directional pleiotropy and the weighted median method provided consistent estimates, residual confounding cannot be completely excluded. Specifically, the absence of subgroup analysis was due to limited availability of comprehensive clinical data for participants. As a result, our study does not investigate possible sex-specific effects of glucocorticoid use on the risk of pancreatitis. Furthermore, the study population consisted predominantly of individuals with European descendants (34–39), potentially limiting generalizability across diverse ethnic backgrounds such as African populations. It is important to note that possible potential genetic heterogeneity within the European population may also affect the validity of our genetic instruments and MR findings. Therefore, further research with larger sample sizes, more genetically diverse populations or ethnic groups, more detailed sex-stratified analyses and longitudinal follow-up is imperative to conclusively validate the causal relationship between glucocorticoid use and pancreatitis risk. Although our selected genetic variants have been rigorously assessed for robustness and independence, it is acknowledged that they may not capture the entirety of glucocorticoid exposure. Future studies could consider expanding the range of genetic tools or incorporating alternative methodological approaches to comprehensively capture the complexity of glucocorticoid use. Despite the fact that our Mendelian randomization analysis effectively mitigates confounding by measured covariates and is sufficiently powered to detect moderate to large effects, it may not be sensitive enough to identify smaller effect sizes. In addition, unmeasured or residual confounders, such as a Western diet or diabetes, may have influenced our results. Another potential limitation of our study is the possibility of type II error. Type II error occurs when the study fails to detect a true effect due to insufficient statistical power. Given the complexity and multifactorial nature of the etiology of pancreatitis, it is possible that our non-significant results may have been influenced by type II error. Future studies with larger sample sizes and more comprehensive data may help to mitigate this issue and provide a clearer understanding of the relationship between glucocorticoid use and pancreatitis risk. Moreover, our study does not consider possible interactions between glucocorticoid use and other medications or treatments that may affect the risk of developing pancreatitis. For instance, glucocorticoids and non-steroidal anti-inflammatory drugs (NSAIDs) are often used together, especially for conditions involving inflammation and pain (8, 105, 106). However, there are numerous case reports linking NSAIDs such as indomethacin, piroxicam, ketoprofen, naproxen, rofecoxib and celecoxib with acute pancreatitis (107). Interestingly, naproxen is often considered the preferred analgesic to limit the risk of developing acute pancreatitis (107). Studies have also suggested that widespread prophylactic use of NSAIDs may significantly reduce the risk of acute pancreatitis following therapeutic endoscopic retrograde cholangiopancreatography (ERCP) (107–109). Post-ERCP pancreatitis is a known complication, and glucocorticoids have been investigated for their potential role in preventing this condition (30). Some studies suggest that glucocorticoids may reduce inflammation and edema, potentially decreasing the incidence of post-ERCP pancreatitis (30). However, the evidence is mixed and sometimes contradictory, suggesting that more research is needed to establish their effectiveness in this setting (30, 108, 110–112). Future research should consider these interactions between glucocorticoid use and other medications or treatments to provide a more comprehensive understanding of pancreatitis risk. In addition, the ability of our study to detect small but clinically significant effects may be limited by several factors, most notably the limited number of SNPs used as instrumental variables (IVs). In Mendelian randomization (MR) studies, statistical power is highly dependent on both the strength and number of IVs (113–115). The limited number of SNPs in this study may reduce the ability to detect associations between the IVs and the exposure variable, which may explain the non-significant results. The minimum detectable effect size (MDES) is also crucial; a study with limited power may fail to detect small but meaningful effects (114, 116). To address this concern, we carried out additional replicated MR analyses using GWAS summary datasets from the FinnGen Biobank Consortium and East Asian descendants, in addition to the original European ancestry GWAS data. The consistent results across these different datasets suggest a degree of clinical significance and increase the credibility of our findings. Moreover, the MR-Egger method is designed to detect and correct for directional pleiotropy, which occurs when genetic variants influence outcome through pathways other than the exposure of interest. The key assumption of MR-Egger is the Instrument Strength Independent of Direct Effect (InSIDE) assumption, which states that the strength of the association of the genetic instrument with the exposure is independent of its direct effect on the outcome (115–117). However, this assumption may not always hold in practice, potentially leading to biased estimates. For instance, if the genetic variants have pleiotropic effects that are not independent of their associations with exposure, the MR-Egger intercept test may indicate the presence of pleiotropy even when it is absent, or fail to detect it when it is present (115–118). This may complicate the interpretation of causal estimates derived from MR-Egger analysis. Also, MR-Egger has less statistical power than other MR methods, such as inverse variance weighted (IVW) regression, especially when the number of genetic variants is small or the genetic instruments are weak (115–117, 119). This reduced power can lead to wider confidence intervals and less precise estimates of the causal effect, which should be taken into account when interpreting the results (114, 120). In our study, the MR-Egger intercept test showed no significant evidence of directional pleiotropy (all P values > 0.05), suggesting that pleiotropy is unlikely to significantly bias our causal estimates. Nevertheless, the limitations of MR-Egger, including its reduced precision, must be acknowledged. To address these limitations and validate the robustness of our findings, we conducted several sensitivity analyses, including the Cochran’s Q test for heterogeneity and the MR-PRESSO method to detect and correct for pleiotropic outliers. These additional analyses help to provide a more comprehensive assessment of the potential bias due to pleiotropy and increase the transparency and reliability of our results (114, 117, 119, 120).

In conclusion, although our MR-Egger results suggest minimal pleiotropic bias, the inherent limitations of this method and the assumptions upon which it is based must be explicitly acknowledged. To improve the power of future studies and mitigate the inherent limitations of the MR-Egger method, it is essential to increase sample sizes and identify stronger genetic tools. Larger sample sizes can improve the ability to detect associations, thereby increasing the overall power of the study (114, 120). In addition, identifying and using multiple stronger genetic variants as IVs can strengthen the instruments and improve the precision of the estimates, thereby reducing bias and increasing power (115, 116). These strategies are essential to accurately assess the causal relationship between glucocorticoid use and the risk of pancreatitis. These limitations should be taken into account when interpreting the results.

Gene-environment interactions occur when environmental factors such as smoking, diet and concomitant medication use interact with genetic predispositions to influence disease risk (121). For example, oxidative stress from alcohol and smoking may exacerbate genetic mutations associated with pancreatitis, such as those in the SPINK1 and CFTR genes (122). Research suggests that genetic variants may influence how individuals respond to environmental factors (121). Thus, epigenetic modifications induced by environmental exposures may affect the expression of genes involved in glucocorticoid metabolism and stress responses, further complicating the relationship between glucocorticoid use and pancreatitis (123). Future research should focus on identifying specific gene-environment interactions that contribute to the risk of pancreatitis in glucocorticoid users. This can be achieved through genome-wide association studies (GWAS) and epigenome-wide association studies (EWAS), which examine the combined effects of genetic variants and environmental factors on disease risk. Such studies should involve large, diverse populations to capture a wide range of genetic and environmental exposures, thereby increasing the generalizability of the findings.

Additionally, our findings have significant implications for healthcare policy regarding glucocorticoid administration and pancreatitis management. Given the widespread prescription of glucocorticoids and the serious consequences associated with pancreatitis development, establishing a definitive causal link is crucial for establishing public health strategies towards early prevention and intervention efforts. Despite the fact that our study found no evidence of an association between glucocorticoid use and an increased incidence of acute pancreatitis, clinicians must remain vigilant when prescribing these drugs because of their well-documented side effects. Healthcare providers should assess the risk-benefit profile of glucocorticoid therapy on a case-by-case basis, particularly in patients with additional risk factors for pancreatitis. Standard preventive measures for pancreatitis should continue to be used in clinical practice. Encouraging lifestyle changes, such as maintaining a healthy diet, regular exercise and avoiding excessive alcohol consumption, is crucial for overall health and may indirectly reduce the risk of pancreatitis in patients with complex medical histories (49, 124, 125). Our findings suggest that routine screening for pancreatitis in glucocorticoid users may not be warranted. However, clinicians should remain vigilant for pancreatitis symptoms in patients with multiple risk factors, particularly those with pre-existing conditions that predispose them to pancreatitis. Based on our findings, future guidelines for glucocorticoid therapy should emphasize targeted monitoring rather than broad screening. Although our study found no statistically significant association between glucocorticoid use and the risk of pancreatitis, even a small increase in risk could raise public health concerns due to the widespread use of these drugs and the potential severity of pancreatitis (1, 2, 8).

Given the high prevalence of glucocorticoid use (8), the absolute number of people affected could be substantial. Although our MR study does not support an association between glucocorticoid use and an increased incidence of acute pancreatitis, vigilant clinical practice and adherence to guidelines are essential to mitigate other potential risks. Glucocorticoid-induced pancreatitis can lead to serious complications, including systemic inflammatory response syndrome (SIRS), multiple organ failure and increased mortality (22, 46). For example, Iqbal et al. (98) reported a case of steroid-induced pancreatitis in a patient receiving high-dose steroids for optic neuritis, highlighting the importance of clinician vigilance. Similarly, Ataallah et al. (17) highlighted the diagnostic challenges of glucocorticoid-induced pancreatitis, particularly in patients with multiple risk factors. These cases suggest that although the incidence may be low, the clinical outcomes can be severe, highlighting the need for a public health strategy to mitigate the risks.

From a patient management perspective, it is important to identify high-risk individuals and monitor them closely during glucocorticoid therapy. Clinicians should exercise caution and carefully weigh the benefits of glucocorticoid therapy against the potential risk of pancreatitis, especially when prescribing glucocorticoids to patients with known risk factors such as a history of pancreatitis, alcohol use or metabolic disorders (17, 126). In these high-risk patients, regular monitoring of pancreatic function and prompt treatment of early symptoms may help prevent severe pancreatitis. Previous studies (21) have shown that glucocorticoid-induced pancreatitis can develop in a dose-dependent manner, suggesting that reducing the dose and duration of glucocorticoid therapy may reduce the risk. In addition, glucocorticoids are associated with a number of other adverse effects, including hyperglycemia, hypertension, osteoporosis, neuropsychiatric adverse effects and immunosuppression (127–130).

As an example, a systematic review and meta-analysis found an increased risk of cataract and glaucoma in patients using systemic glucocorticoids (127). Another study reported significant associations between short-term systemic glucocorticoid use and an increased risk of infection and hyperglycemia (128). Understanding the mechanisms underlying these glucocorticoid-induced adverse effects is essential for the development of safer medication strategies. The implementation of regular monitoring, dose reduction, shorter duration of therapy and, where appropriate, alternative treatments in high-risk patients may also help to reduce these risks (129, 131–133). In addition, our study uses Mendelian randomization (MR) to investigate the causal relationship between glucocorticoid use and the risk of pancreatitis. This approach helps to control for confounding while providing more robust evidence of causality (41, 102–104).

The application of MR to the understanding of glucocorticoid-related adverse effects may facilitate the development of targeted mitigation strategies to improve patient outcomes. Further research is needed to identify biomarkers that predict susceptibility to glucocorticoid-related adverse effects and to develop targeted interventions. Studies using pharmacogenomic approaches may provide insight into individual variability in response to glucocorticoid therapy. We recommend that future research should focus on the development and validation of risk assessment tools that integrate genetic, clinical and lifestyle factors to identify patients at high risk of glucocorticoid-related pancreatitis and thus develop safer drug use strategies.

## 5 Conclusion

This study represents the first MR investigating the causal relationship between glucocorticoid use and pancreatitis. However, our MR results do not provide evidence, supporting an association between glucocorticoid use and increased incidence of pancreatitis.

## Data Availability

The datasets presented in this study can be found in online repositories. The names of the repository/repositories and accession number(s) can be found in the article/**Supplementary Material**.

## References

[1] Lankisch PG Apte M Banks PA. Acute pancreatitis. Lancet (London England). (2015) 386:85–96. doi: 10.1016/S0140-6736(14)60649-8 25616312

[2] Mederos MA Reber HA Girgis MD. Acute pancreatitis: A review. Jama. (2021) 325:382–90. doi: 10.1001/jama.2020.20317 33496779

[3] Iannuzzi JP King JA Leong JH Quan J Windsor JW Tanyingoh D. Global incidence of acute pancreatitis is increasing over time: A systematic review and meta-analysis. Gastroenterology. (2022) 162:122–34. doi: 10.1053/j.gastro.2021.09.043 34571026

[4] Oskarsson V Mehrabi M Orsini N Hammarqvist F Segersvärd R Andrén-Sandberg A. Validation of the harmless acute pancreatitis score in predicting nonsevere course of acute pancreatitis. Pancreatology. (2011) 11:464–8. doi: 10.1159/000331502 21968430

[5] Lankisch PG Breuer N Bruns A Weber-Dany B Lowenfels AB Maisonneuve P. Natural history of acute pancreatitis: a long-term population-based study. Am J gastroenterology. (2009) 104:2797–805; quiz 806. doi: 10.1038/ajg.2009.405 19603011

[6] Vinklerová I Procházka M Procházka V Urbánek K. Incidence, severity, and etiology of drug-induced acute pancreatitis. Digestive Dis Sci. (2010) 55:2977–81. doi: 10.1007/s10620-010-1277-3 20499176

[7] Spanier BW Tuynman HA van der Hulst RW Dijkgraaf MG Bruno MJ. Acute pancreatitis and concomitant use of pancreatitis-associated drugs. Am J gastroenterology. (2011) 106:2183–8. doi: 10.1038/ajg.2011.303 21912439

[8] Wallace BI Tsai HJ Lin P Aasbjerg K Wu AC Tsai YF. Prevalence and prescribing patterns of oral corticosteroids in the United States, Taiwan, and Denmark, 2009-2018. Clin Trans science. (2023) 16:2565–76. doi: 10.1111/cts.13649 PMC10719491 37718472

[9] Schäcke H Döcke WD Asadullah K. Mechanisms involved in the side effects of glucocorticoids. Pharmacol Ther. (2002) 96:23–43. doi: 10.1016/S0163-7258(02)00297-8 12441176

[10] Oray M Abu Samra K Ebrahimiadib N Meese H Foster CS. Long-term side effects of glucocorticoids. Expert Opin Drug safety. (2016) 15:457–65. doi: 10.1517/14740338.2016.1140743 26789102

[11] Wang-Liang C Reid S Barkley J Jain P. Methylprednisolone-induced acute pancreatitis, a case presentation. Discovery Med. (2022) 34:79–81. 36281028

[12] Iqbal K Rathore SS Hanyalu Shankar V Deepika K Pattan V Koritala T. A case of acute pancreatitis in a patient receiving high-dose steroids for optic neuritis. Cureus. (2021) 13:e19132. doi: 10.7759/cureus.19132 34858765 PMC8614170

[13] Sadr-Azodi O Mattsson F Bexlius TS Lindblad M Lagergren J Ljung R. Association of oral glucocorticoid use with an increased risk of acute pancreatitis: a population-based nested case-control study. JAMA Internal Med. (2013) 173:444–9. doi: 10.1001/jamainternmed.2013.2737 23440105

[14] Nango D Hirose Y Goto M Echizen H. Analysis of the Association of Administration of various glucocorticoids with development of acute pancreatitis using US Food and Drug Administration adverse event reporting system (FAERS). J Pharm Health Care Sci. (2019) 5:5. doi: 10.1186/s40780-019-0134-6 30858980 PMC6394067

[15] Richard K Waggoner G Donnan M Ayesu K Madruga M Carlan SJ. Epidural steroid injection-induced pancreatitis: A case report. Am J Case Rep. (2020) 21:e921241. doi: 10.12659/AJCR.921241 32037393 PMC7032528

[16] Minupuri A Patel R Alam F Rather M Baba RH. Steroid-induced pancreatitis: establishing an accurate association poses a challenge. Cureus. (2020) 12:e9589. doi: 10.7759/cureus.9589 32923195 PMC7478484

[17] Ataallah B Abdulrahman M Al-Zakhari R Buttar BS Nabeel S. Steroid-induced pancreatitis: A challenging diagnosis. Cureus. (2020) 12:e8939. doi: 10.7759/cureus.8939 32765985 PMC7398688

[18] Yahiaoui N Roche M Aissaoui-Hoffmann N Keita BA Mallaret M. Intravenous methylprednisolone induced acute pancreatitis. Eur J Clin Pharmacol. (2017) 73:645–6. doi: 10.1007/s00228-017-2207-5 28132081

[19] Sabre A Guthrie MM Maleknia R. Acute necrotising pancreatitis derived from low-dose corticosteroid use: an important reminder of clinical management. BMJ Case Rep. (2015) 2015:bcr2015209325. doi: 10.1136/bcr-2015-209325 PMC4493234 26150628

[20] Ungprasert P Permpalung N Summachiwakij S Manatsathit W. A case of recurrent acute pancreatitis due to intra-articular corticosteroid injection. JOP: J Pancreas. (2014) 15:208–9. doi: 10.6092/1590-8577/2214 24618449

[21] Yoshizawa Y Ogasa S Izaki S Kitamura K. Corticosteroid-induced pancreatitis in patients with autoimmune bullous disease: case report and prospective study. Dermatol (Basel Switzerland). (1999) 198:304–6. doi: 10.1159/000018137 10393460

[22] Wolfe D Kanji S Yazdi F Barbeau P Rice D Beck A. Drug induced pancreatitis: A systematic review of case reports to determine potential drug associations. PloS One. (2020) 15:e0231883. doi: 10.1371/journal.pone.0231883 32302358 PMC7164626

[23] Weissman S Aziz M Perumpail RB Mehta TI Patel R Tabibian JH. Ever-increasing diversity of drug-induced pancreatitis. World J gastroenterology. (2020) 26:2902–15. doi: 10.3748/wjg.v26.i22.2902 PMC7304112 32587438

[24] Steinberg WM Lewis JH. Steroid-induced pancreatitis: does it really exist? Gastroenterology. (1981) 81:799–808. doi: 10.1016/0016-5085(81)90511-4 7021304

[25] Hung WY Abreu Lanfranco O. Contemporary review of drug-induced pancreatitis: A different perspective. World J gastrointestinal pathophysiology. (2014) 5:405–15. doi: 10.4291/wjgp.v5.i4.405 PMC4231505 25400984

[26] Blomgren KB Sundström A Steineck G Genell S Sjöstedt S Wiholm BE. A Swedish case-control network for studies of drug-induced morbidity–acute pancreatitis. Eur J Clin Pharmacol. (2002) 58:275–83. doi: 10.1007/s00228-002-0471-4 12136374

[27] Makol A Petri M. Pancreatitis in systemic lupus erythematosus: frequency and associated factors - a review of the Hopkins Lupus Cohort. J Rheumatol. (2010) 37:341–5. doi: 10.3899/jrheum.090829 20032096

[28] Chawla S Atten MJ Attar BM. Acute pancreatitis as a rare initial manifestation of Wegener's granulomatosis. A case based review of literature. JOP: J pancreas. (2011) 12:167–9. 21386646

[29] Derk CT DeHoratius RJ. Systemic lupus erythematosus and acute pancreatitis: a case series. Clin Rheumatol. (2004) 23:147–51. doi: 10.1007/s10067-003-0793-3 15045630

[30] Weiner GR Geenen JE Hogan WJ Catalano MF. Use of corticosteroids in the prevention of post-ERCP pancreatitis. Gastrointestinal endoscopy. (1995) 42:579–83. doi: 10.1016/S0016-5107(95)70014-5 8674931

[31] Zheng J Yang QJ Dang FT Yang J. Drug-induced pancreatitis: An update. Arab J gastroenterology: Off Publ Pan-Arab Assoc Gastroenterology. (2019) 20:183–8. doi: 10.1016/j.ajg.2019.11.005 31806409

[32] Burgess S Daniel RM Butterworth AS Thompson SG. Network Mendelian randomization: using genetic variants as instrumental variables to investigate mediation in causal pathways. Int J Epidemiol. (2015) 44:484–95. doi: 10.1093/ije/dyu176 PMC4469795 25150977

[33] Lawlor DA. Commentary: Two-sample Mendelian randomization: opportunities and challenges. Int J Epidemiol. (2016) 45:908–15. doi: 10.1093/ije/dyw127 PMC5005949 27427429

[34] Sakaue S Kanai M Tanigawa Y Karjalainen J Kurki M Koshiba S. A cross-population atlas of genetic associations for 220 human phenotypes. Nat Genet. (2021) 53:1415–24. doi: 10.1038/s41588-021-00931-x PMC12208603 34594039

[35] Zhu Y Zhuang Z Lv J Sun D Pei P Yang L. A genome-wide association study based on the China Kadoorie Biobank identifies genetic associations between snoring and cardiometabolic traits. Commun Biol. (2024) 7:305. doi: 10.1038/s42003-024-05978-0 38461358 PMC10924953

[36] Xue A Zhu Z Wang H Jiang L Visscher PM Zeng J. Unravelling the complex causal effects of substance use behaviours on common diseases. Commun Med. (2024) 4:43. doi: 10.1038/s43856-024-00473-3 38472333 PMC10933313

[37] Mbatchou J Barnard L Backman J Marcketta A Kosmicki JA Ziyatdinov A. Computationally efficient whole-genome regression for quantitative and binary traits. Nat Genet. (2021) 53:1097–103. doi: 10.1038/s41588-021-00870-7 34017140

[38] Zhang Y Yu J Pei H Zhao X Wang C Wang G. Potential causal associations of PM2.5 and osteoporosis: a two-sample mendelian randomization study. Front Genet. (2024) 15:1263916. doi: 10.3389/fgene.2024.1263916 38463167 PMC10921569

[39] Loh PR Kichaev G Gazal S Schoech AP Price AL. Mixed-model association for biobank-scale datasets. Nat Genet. (2018) 50:906–8. doi: 10.1038/s41588-018-0144-6 PMC6309610 29892013

[40] Burgess S Scott RA Timpson NJ Davey Smith G Thompson SG. Using published data in Mendelian randomization: a blueprint for efficient identification of causal risk factors. Eur J Epidemiol. (2015) 30:543–52. doi: 10.1007/s10654-015-0011-z PMC4516908 25773750

[41] Bowden J Davey Smith G Burgess S. Mendelian randomization with invalid instruments: effect estimation and bias detection through Egger regression. Int J Epidemiol. (2015) 44:512–25. doi: 10.1093/ije/dyv080 PMC4469799 26050253

[42] Bowden J Davey Smith G Haycock PC Burgess S. Consistent estimation in mendelian randomization with some invalid instruments using a weighted median estimator. Genet Epidemiol. (2016) 40:304–14. doi: 10.1002/gepi.21965 PMC4849733 27061298

[43] Burgess S Butterworth A Thompson SG. Mendelian randomization analysis with multiple genetic variants using summarized data. Genet Epidemiol. (2013) 37:658–65. doi: 10.1002/gepi.21758 PMC4377079 24114802

[44] Verbanck M Chen CY Neale B Do R. Detection of widespread horizontal pleiotropy in causal relationships inferred from Mendelian randomization between complex traits and diseases. Nat Genet. (2018) 50:693–8. doi: 10.1038/s41588-018-0099-7 PMC6083837 29686387

[45] Lin SH Brown DW Machiela MJ. LDtrait: an online tool for identifying published phenotype associations in linkage disequilibrium. Cancer Res. (2020) 80:3443–6. doi: 10.1158/0008-5472.CAN-20-0985 PMC7442674 32606005

[46] Szatmary P Grammatikopoulos T Cai W Huang W Mukherjee R Halloran C. Acute pancreatitis: diagnosis and treatment. Drugs. (2022) 82:1251–76. doi: 10.1007/s40265-022-01766-4 PMC9454414 36074322

[47] Gardner TB Adler DG Forsmark CE Sauer BG Taylor JR Whitcomb DC. ACG clinical guideline: chronic pancreatitis. Off J Am Coll Gastroenterology| ACG. (2020) 115:322–39. doi: 10.14309/ajg.0000000000000535 32022720

[48] Leppäniemi A Tolonen M Tarasconi A Segovia-Lohse H Gamberini E Kirkpatrick AW. 2019 WSES guidelines for the management of severe acute pancreatitis. World J Emergency Surg. (2019) 14:1–20. doi: 10.1186/s13017-019-0247-0 PMC6567462 31210778

[49] Greenberg JA Hsu J Bawazeer M Marshall J Friedrich JO Nathens A. Clinical practice guideline: management of acute pancreatitis. Can J surgery. (2016) 59:128. doi: 10.1503/cjs.015015 PMC4814287 27007094

[50] Andersen V Sonne J Andersen M. Spontaneous reports on drug-induced pancreatitis in Denmark from 1968 to 1999. Eur J Clin Pharmacol. (2001) 57:517–21. doi: 10.1007/s002280100346 11699619

[51] Fagenholz PJ Castillo CF Harris NS Pelletier AJ Camargo CA Jr. Increasing United States hospital admissions for acute pancreatitis, 1988-2003. Ann Epidemiol. (2007) 17:491–7. doi: 10.1016/j.annepidem.2007.02.002 17448682

[52] Wang M Jiang Z Liang H. Glucocorticoids in acute pancreatitis: a propensity score matching analysis. BMC gastroenterology. (2021) 21:331. doi: 10.1186/s12876-021-01907-1 34433425 PMC8386156

[53] Hoffman BI Katz WA. The gastrointestinal manifestations of systemic lupus erythematosus: a review of the literature. Semin Arthritis rheumatism. (1980) 9:237–47. doi: 10.1016/0049-0172(80)90016-5 6996096

[54] Rünzi M Layer P. Drug-associated pancreatitis: facts and fiction. Pancreas. (1996) 13:100–9. doi: 10.1097/00006676-199607000-00014 8783341

[55] Bencosme SA Lazarus SS. The pancreas of cortisone-treated rabbits; pathogenic study. AMA Arch pathology. (1956) 62:285–95. 13361671

[56] Dreiling DA Janowitz HD Rolbin H. Effect of ACTH and adrenocortical steroids on external pancreatic secretion in man. New Engl J Med. (1958) 258:603–5. doi: 10.1056/NEJM195803202581207 13517525

[57] Nelp WB. Acute pancreatitis associated with steroid therapy. Arch Internal Med. (1961) 108:702–10. doi: 10.1001/archinte.1961.03620110042007 14478884

[58] Stumpf HH Wilens SL Somoza C. Pancreatic lesions and peripancreatic fat necrosis in cortisone-treated rabbits. Lab investigation; J Tech Methods pathology. (1956) 5:224–35. 13296385

[59] Cameron JL Capuzzi DM Zuidema GD Margolis S. Acute pancreatitis with hyperlipemia: the incidence of lipid abnormalities in acute pancreatitis. Ann surgery. (1973) 177:483–9. doi: 10.1097/00000658-197304000-00017 PMC1355660 4691868

[60] Melby JC. Drug spotlight program: systemic corticosteroid therapy: pharmacology and endocrinologic considerations. Ann Internal Med. (1974) 81:505–12. doi: 10.7326/0003-4819-81-4-505 4137614

[61] Franckson JR Gepts W Bastenie PA Conard V Cordier N Kovacs L. [Observations on the experimental steroid diabetes in rats]. Acta endocrinologica. (1953) 14:153–69. doi: 10.1530/acta.0.0140153 13113836

[62] Hausberger FX Ramsay AJ. Steroid diabetes in Guinea pigs; effects of cortisone administration on blood- and urinary glucose, nitrogen excretion, fat deposition, and the islets of Langerhans. Endocrinology. (1953) 53:423–35. doi: 10.1210/endo-53-4-423 13095355

[63] Abelove WA Paschkis KE. Comparison of the diabetogenic action of cortisone and growth hormone in different species. Endocrinology. (1954) 55:637–54. doi: 10.1210/endo-55-5-637 13210306

[64] Hinton JW Pfeffer RB. Some relationships between adrenal medullary and cortical substances and exocrine function of the pancreas in man. Gastroenterology. (1956) 31:746–57. doi: 10.1016/S0016-5085(19)35816-0 13397699

[65] Nelp WB Banwell JG Hendrix TR. Pancreatic function and the viscosity of pancreatic juice before and during cortisone administration. Bull Johns Hopkins Hospital. (1961) 109:292–301. 14478883

[66] Sircus W. The effect of corticotrophin and corticosteroids on the external secretion of the pancreas in dogs. Gut. (1961) 2:338–45. doi: 10.1136/gut.2.4.338 PMC1413359 13913433

[67] Tiscornia OM Hansky J Janowitz HD Dreiling DA. The adrenal cortex and external pancreatic secretion in the dog. J Mount Sinai Hospital New York. (1965) 32:551–61. 5212656

[68] Kimura T Zuidema GD Cameron JL. Steroid administration and acute pancreatitis: studies with an isolated, perfused canine pancreas. Surgery. (1979) 85:520–4. 432812

[69] Berry AR Taylor TV. Effect of drugs on the pulmonary changes in experimental acute pancreatitis in the rat. Gut. (1982) 23:481–4. doi: 10.1136/gut.23.6.481 PMC1419705 7076022

[70] Takaoka K Kataoka K Sakagami J. The effect of steroid pulse therapy on the development of acute pancreatitis induced by closed duodenal loop in rats. J gastroenterology. (2002) 37:537–42. doi: 10.1007/s005350200083 12162412

[71] Zhang XP Zhang L Wang Y Cheng QH Wang JM Cai W. Study of the protective effects of dexamethasone on multiple organ injury in rats with severe acute pancreatitis. JOP: J pancreas. (2007) 8:400–12. 17625291

[72] Schepers NJ Besselink MG van Santvoort HC Bakker OJ Bruno MJ. Early management of acute pancreatitis. Best Pract Res Clin gastroenterology. (2013) 27:727–43. doi: 10.1016/j.bpg.2013.08.007 24160930

[73] Balk RA. Systemic inflammatory response syndrome (SIRS): where did it come from and is it still relevant today? Virulence. (2014) 5:20–6. doi: 10.4161/viru.27135 PMC3916374 24280933

[74] de Leeuw K Niemeijer AS Eshuis J Nieuwenhuis MK Beerthuizen GI Janssen WM. Effect and mechanism of hydrocortisone on organ function in patients with severe burns. J Crit Care. (2016) 36:200–6. doi: 10.1016/j.jcrc.2016.06.007 27546772

[75] Meduri GU Bridges L Shih MC Marik PE Siemieniuk RAC Kocak M. Prolonged glucocorticoid treatment is associated with improved ARDS outcomes: analysis of individual patients' data from four randomized trials and trial-level meta-analysis of the updated literature. Intensive Care Med. (2016) 42:829–40. doi: 10.1007/s00134-015-4095-4 26508525

[76] Wang K Tan F Zhou R Liu D Ni Z Liu J. Therapeutic response to corticosteroids in a critically ill patient with COVID-19: A case report. Medicine. (2020) 99:e21597. doi: 10.1097/MD.0000000000021597 32756215 PMC7402741

[77] Studley JG Schenk WG Jr. Pathophysiology of acute pancreatitis: evaluation of the effect and mode of action of steroids in experimental pancreatitis in dogs. Am J surgery. (1982) 143:761–4. doi: 10.1016/0002-9610(82)90054-X 7091513

[78] Yu WQ Zhang SY Fu SQ Fu QH Lu WN Zhang J. Dexamethasone protects the glycocalyx on the kidney microvascular endothelium during severe acute pancreatitis. J Zhejiang Univ Sci B. (2019) 20:355–62. doi: 10.1631/jzus.B1900006 PMC6454317 30932380

[79] Okanishi H Nagata T Nakane S Watari T. Comparison of initial treatment with and without corticosteroids for suspected acute pancreatitis in dogs. J small Anim practice. (2019) 60:298–304. doi: 10.1111/jsap.12994 30868606

[80] Yu M Yang Z Zhu Y Lu N. Efficacy of glucocorticoids in rodents of severe acute pancreatitis: a meta-analysis. Int J Clin Exp pathology. (2014) 7:3647–61. PMC4128976 25120741

[81] Zhao S Yang J Liu T Zeng J Mi L Xiang K. Dexamethasone inhibits NF−кBp65 and HMGB1 expression in the pancreas of rats with severe acute pancreatitis. Mol Med Rep. (2018) 18:5345–52. doi: 10.3892/mmr PMC6236277 30365121

[82] Barnes PJ. Anti-inflammatory actions of glucocorticoids: molecular mechanisms. Clin Sci (London England: 1979). (1998) 94:557–72. doi: 10.1042/cs0940557 9854452

[83] Santos AA Scheltinga MR Lynch E Brown EF Lawton P Chambers E. Elaboration of interleukin 1-receptor antagonist is not attenuated by glucocorticoids after endotoxemia. Arch Surg (Chicago Ill: 1960). (1993) 128:138–43; discussion 43-4. doi: 10.1001/archsurg.1993.01420140015003 8431115

[84] Yue M Li C Zhao E Li X. [The effect of anisodaminum and dexamethasone on microcirculation, TNF, LPO and pathology in MODS]. Zhonghua wai ke za zhi [Chinese J surgery]. (1997) 35:392–4. 10677970

[85] Liu J Wei X Fu J Liu J Yuan Y Wu Y. Stady of the relationship among endothelin, nitric oxide, oxgen free radical and acute pancreatitis. Zhongguo Yishi Zazhi. (2003) 5:28–9.

[86] Natanson C Hoffman WD Suffredini AF Eichacker PQ Danner RL. Selected treatment strategies for septic shock based on proposed mechanisms of pathogenesis. Ann Internal Med. (1994) 120:771–83. doi: 10.7326/0003-4819-120-9-199405010-00009 8147551

[87] Meduri GU. New rationale for glucocorticoid treatment in septic shock. J chemotherapy (Florence Italy). (1999) 11:541–50. doi: 10.1179/joc.1999.11.6.541 10678798

[88] Lanza L Scudeletti M Monaco E Monetti M Puppo F Filaci G. Possible differences in the mechanism(s) of action of different glucocorticoid hormone compounds. Ann New York Acad Sci. (1999) 876:193–7. doi: 10.1111/j.1749-6632.1999.tb07638.x 10415609

[89] Liu QG Xu GF Geng ZM Liu XM Zhang T. Effects of dexamethasone on apoptosis of pancreatic acinar cells in severe acute pancreatitis in rats. Xi'an jiao tong da xue xue bao Yi xue ban. (2003) 1:56.

[90] Lasa M Brook M Saklatvala J Clark AR. Dexamethasone destabilizes cyclooxygenase 2 mRNA by inhibiting mitogen-activated protein kinase p38. Mol Cell Biol. (2001) 21:771–80. doi: 10.1128/MCB.21.3.771-780.2001 PMC86669 11154265

[91] Yang Z Liu D Wang X Zhang X Zhao X Li M. Experimental study on the treatment of acute necrotizing pancreatitis by dexamethasone. Chin J Bases Clin Gen Surg. (2002) 9:26–7.

[92] Shimosegawa T. Are glucocorticoids really useful for the treatment of acute pancreatitis? J Gastroenterol. (2002) 37:580–1. doi: 10.1007/s005350200092 12162421

[93] Cai O Tan S Zhao S Yang J Liu T Zeng J. From Pathogenesis, Clinical Manifestation, and Diagnosis to Treatment: An Overview on Autoimmune Pancreatitis Dexamethasone inhibits NF−кBp65 and HMGB1 expression in the pancreas of rats with severe acute pancreatitis. Gastroenterol Res Pract. (2017) 2017:3246459. 10.1155/2017/3246459 PMC5288542 28197205

[94] Stephenson HE Jr. Pfeffer RB Saypol GM. Acute hemorrhagic pancreatitis; report of a case with cortisone treatment. AMA Arch surgery. (1952) 65:307–8. doi: 10.1001/archsurg.1952.01260020320013 14943366

[95] Wan MH Li J Gong HL Xue P Zhu L Chen GY. Clinical observation on the effect of dexamethasone and Chinese herbal decoction for purgation in severe acute pancreatitis patients. Chin J Integr Med. (2011) 17:141–5. doi: 10.1007/s11655-011-0630-5 21390581

[96] Dong LH Liu ZM Wang SJ Zhao SJ Zhang D Chen Y. Corticosteroid therapy for severe acute pancreatitis: a meta-analysis of randomized, controlled trials. Int J Clin Exp pathology. (2015) 8:7654–60. PMC4555660 26339332

[97] Nango D Hirose Y Goto M Echizen H Okanishi H Nagata T. Analysis of the Association of Administration of various glucocorticoids with development of acute pancreatitis using US Food and Drug Administration adverse event reporting system (FAERS) Comparison of initial treatment with and without corticosteroids for suspected acute pancreatitis in dogs. J Pharm Health Care Sci. (2019) 5:5. doi: 10.1186/s40780-019-0134-6 30858980 PMC6394067

[98] Iqbal K Rathore SS Hanyalu Shankar V Deepika K Pattan V Koritala T. A case of acute pancreatitis in a patient receiving high-dose steroids for optic neuritis consequences of COVID-19 for the pancreas. Cureus. (2021) 13:e19132. doi: 10.7759/cureus.19132 34858765 PMC8614170

[99] Shi AX Zivich PN Chu H. A comprehensive review and tutorial on confounding adjustment methods for estimating treatment effects using observational data. Appl Sci. (2024) 14(9):3662. doi: 10.3390/app14093662

[100] Braga LH Farrokhyar F Bhandari M. Confounding: what is it and how do we deal with it? Can J Surg J canadien chirurgie. (2012) 55:132–8. doi: 10.1503/cjs PMC3310769 22564517

[101] Smith CJ. Methods to account for confounding in observational studies. Phlebology. (2011) 26:125–7. doi: 10.1258/phleb.2011.011j01 21471584

[102] Chen X Kong J Diao X Cai J Zheng J Xie W. Depression and prostate cancer risk: A Mendelian randomization study. Cancer Med. (2020) 9:9160–7. doi: 10.1002/cam4.3493 PMC7724297 33027558

[103] Skrivankova VW Richmond RC Woolf BAR Yarmolinsky J Davies NM Swanson SA. Strengthening the reporting of observational studies in epidemiology using mendelian randomization: the STROBE-MR statement. Jama. (2021) 326:1614–21. doi: 10.1001/jama.2021.18236 34698778

[104] Sekula P Del Greco MF Pattaro C Köttgen A. Mendelian randomization as an approach to assess causality using observational data. J Am Soc Nephrology: JASN. (2016) 27:3253–65. doi: 10.1681/ASN.2016010098 PMC5084898 27486138

[105] Garcia Rodríguez LA Hernández-Díaz S. The risk of upper gastrointestinal complications associated with nonsteroidal anti-inflammatory drugs, glucocorticoids, acetaminophen, and combinations of these agents. Arthritis Res. (2001) 3:98–101. doi: 10.1186/ar146 11178116 PMC128885

[106] Ritsmer Stormholt E Steiness J Bauer Derby C Esta Larsen M Maagaard M Mathiesen O. Paracetamol, non-steroidal anti-inflammatory drugs and glucocorticoids for postoperative pain: A protocol for a systematic review with meta-analysis and trial sequential analysis. Acta anaesthesiologica Scandinavica. (2021) 65:1505–13. doi: 10.1111/aas.13943 34138463

[107] Pezzilli R Morselli-Labate AM Corinaldesi R. NSAIDs and acute pancreatitis: A systematic review. Pharm (Basel Switzerland). (2010) 3:558–71. doi: 10.3390/ph3030558 PMC4033969 27713268

[108] Cahyadi O Tehami N de-Madaria E Siau K. Post-ERCP pancreatitis: prevention, diagnosis and management. Medicina (Kaunas Lithuania). (2022) 58(9):1261. doi: 10.3390/medicina58091261 36143938 PMC9502657

[109] Yuhara H Ogawa M Kawaguchi Y Igarashi M Shimosegawa T Mine T. Pharmacologic prophylaxis of post-endoscopic retrograde cholangiopancreatography pancreatitis: protease inhibitors and NSAIDs in a meta-analysis. J gastroenterology. (2014) 49:388–99. doi: 10.1007/s00535-013-0834-x 23720090

[110] Buxbaum JL Freeman M Amateau SK Chalhoub JM Coelho-Prabhu N Desai M. American Society for Gastrointestinal Endoscopy guideline on post-ERCP pancreatitis prevention strategies: summary and recommendations. Gastrointestinal endoscopy. (2023) 97:153–62. doi: 10.1016/j.gie.2022.10.005 36517310

[111] Zheng M Bai J Yuan B Lin F You J Lu M. Meta-analysis of prophylactic corticosteroid use in post-ERCP pancreatitis. BMC gastroenterology. (2008) 8:6. doi: 10.1186/1471-230X-8-6 18271973 PMC2258301

[112] Sherman S Blaut U Watkins JL Barnett J Freeman M Geenen J. Does prophylactic administration of corticosteroid reduce the risk and severity of post-ERCP pancreatitis: a randomized, prospective, multicenter study. Gastrointestinal endoscopy. (2003) 58:23–9. doi: 10.1067/mge.2003.307 12838216

[113] Freeman G Cowling BJ Schooling CM. Power and sample size calculations for Mendelian randomization studies using one genetic instrument. Int J Epidemiol. (2013) 42:1157–63. doi: 10.1093/ije/dyt110 23934314

[114] Burgess S. Sample size and power calculations in Mendelian randomization with a single instrumental variable and a binary outcome. Int J Epidemiol. (2014) 43:922–9. doi: 10.1093/ije/dyu005 PMC4052137 24608958

[115] Pierce BL Ahsan H Vanderweele TJ. Power and instrument strength requirements for Mendelian randomization studies using multiple genetic variants. Int J Epidemiol. (2011) 40:740–52. doi: 10.1093/ije/dyq151 PMC3147064 20813862

[116] Teumer A. Common methods for performing mendelian randomization. Front Cardiovasc Med. (2018) 5:51. doi: 10.3389/fcvm.2018.00051 29892602 PMC5985452

[117] Burgess S Thompson SG. Interpreting findings from Mendelian randomization using the MR-Egger method. Eur J Epidemiol. (2017) 32:377–89. doi: 10.1007/s10654-017-0255-x PMC5506233 28527048

[118] Dai JY Peters U Wang X Kocarnik J Chang-Claude J Slattery ML. Diagnostics for pleiotropy in mendelian randomization studies: global and individual tests for direct effects. Am J Epidemiol. (2018) 187:2672–80. doi: 10.1093/aje/kwy177 PMC6269243 30188971

[119] Lin Z Pan I Pan W. A practical problem with Egger regression in Mendelian randomization. PloS Genet. (2022) 18:e1010166. doi: 10.1371/journal.pgen.1010166 35507585 PMC9109933

[120] Brion MJ Shakhbazov K Visscher PM. Calculating statistical power in Mendelian randomization studies. Int J Epidemiol. (2013) 42:1497–501. doi: 10.1093/ije/dyt179 PMC3807619 24159078

[121] Virolainen SJ VonHandorf A Viel K Weirauch MT Kottyan LC. Gene-environment interactions and their impact on human health. Genes immunity. (2023) 24:1–11. doi: 10.1038/s41435-022-00192-6 36585519 PMC9801363

[122] Garg PK Narayana D. Changing phenotype and disease behaviour of chronic pancreatitis in India: evidence for gene-environment interactions. Global health Epidemiol Genomics. (2016) 1:e17. doi: 10.1017/gheg.2016.13 PMC5870434 29868209

[123] Kubota T Miyake K Hirasawa T. Epigenetic understanding of gene-environment interactions in psychiatric disorders: a new concept of clinical genetics. Clin epigenetics. (2012) 4:1. doi: 10.1186/1868-7083-4-1 22414323 PMC3305338

[124] Arnett DK Blumenthal RS Albert MA Buroker AB Goldberger ZD Hahn EJ. 2019 ACC/AHA guideline on the primary prevention of cardiovascular disease: A report of the american college of cardiology/american heart association task force on clinical practice guidelines. Circulation. (2019) 140:e596–646. doi: 10.1161/CIR.0000000000000678 PMC7734661 30879355

[125] Shimizu K Ito T Irisawa A Ohtsuka T Ohara H Kanno A. Evidence-based clinical practice guidelines for chronic pancreatitis 2021. J gastroenterology. (2022) 57:709–24. doi: 10.1007/s00535-022-01911-6 PMC9522716 35994093

[126] Minupuri A Patel R Alam F Rather M Baba RH Chen X. Steroid-Induced Pancreatitis: Establishing an Accurate Association Poses a Challenge Depression and prostate cancer risk: A Mendelian randomization study. Cureus. (2020) 12:e9589. doi: 10.7759/cureus.9589 32923195 PMC7478484

[127] Black RJ Hill CL Lester S Dixon WG. The association between systemic glucocorticoid use and the risk of cataract and glaucoma in patients with rheumatoid arthritis: A systematic review and meta-analysis. PloS One. (2016) 11:e0166468. doi: 10.1371/journal.pone.0166468 27846316 PMC5112962

[128] Fan HP Zhou Y Zhou Y Jin J Hu TY. Association between short-term systemic use of glucocorticoids and prognosis of cardiogenic shock: a retrospective analysis. BMC anesthesiology. (2023) 23:169. doi: 10.1186/s12871-023-02131-y 37202727 PMC10193317

[129] Barker HL Morrison D Llano A Sainsbury CAR Jones GC. Practical guide to glucocorticoid induced hyperglycaemia and diabetes. Diabetes therapy: research Treat Educ Diabetes related Disord. (2023) 14:937–45. doi: 10.1007/s13300-023-01393-6 PMC10037401 36961675

[130] Koning A van der Meulen M Schaap D Satoer DD Vinkers CH van Rossum EFC. Neuropsychiatric adverse effects of synthetic glucocorticoids: A systematic review and meta-analysis. J Clin Endocrinol Metab. (2024) 109:e1442–e51. doi: 10.1210/clinem/dgad701 PMC11099480 38038629

[131] Liu D Ahmet A Ward L Krishnamoorthy P Mandelcorn ED Leigh R. A practical guide to the monitoring and management of the complications of systemic corticosteroid therapy. Allergy asthma Clin Immunol. (2013) 9:30. doi: 10.1186/1710-1492-9-30 23947590 PMC3765115

[132] Zhao L Zhao A Chen T Chen W Liu J Wei R. Global and targeted metabolomics evidence of the protective effect of chinese patent medicine jinkui shenqi pill on adrenal insufficiency after acute glucocorticoid withdrawal in rats. J Proteome Res. (2016) 15:2327–36. doi: 10.1021/acs.jproteome.6b00409 PMC5614501 27267777

[133] Luypaert A Vanden Berghe W Tavernier J Libert C De Bosscher K. Strategies and Compounds to Circumvent Glucocorticoid-Induced Side Effects. In: Riccardi C Levi-Schaffer F Tiligada E, editors. Immunopharmacology and Inflammation. Springer International Publishing, Cham (2018). p. 283–305.

