# Supplementary material for: The causality between use of glucocorticoids and risk of pancreatitis: a Mendelian randomization study
Source: Front Immunol. 2024 Aug 15;15:1420840. doi: 10.3389/fimmu.2024.1420840 (PMC11363070; doi:10.3389/fimmu.2024.1420840)

Supplementary Material

1 Supplementary Tables

Supplement Table 1. Detailed data of cohort.

Variable	Number of Cases	Number of Controls	Sample size	Data Resource	Population	PMID	Year
AP	3,798	476,104	479,902	ebi-a-GCST90018789	European	34594039	2021
	827	177,471	178,298	ebi-a- GCST90018569	East Asian	34594039	2021
	3,022	195,144	198,166	finn-b-K11_ACUTPANC	European	-	2021
CP	1,424	476,104	477,528	ebi-a-GCST90018821	European	34594039	2021
	457	177,471	177,928	ebi-a-GCST90018601	East Asian	34594039	2021
	1,737	195,144	196,881	finn-b-K11_CHRONPANC	European	-	2021
glucocorticoid usage	17,352	188,348	205,700	ebi-a-GCST90019000	European	34594039	2021
	13,102	165,624	178,726	ebi-a-GCST90018780	East Asian	34594039	2021
Alcohol intake frequency	-	-	462,346	ukb-b-5779	European	-	2018
Body mass index	-	-	532,396	ebi-a-GCST90029007	European	29892013	2018
Cholelithiasis gall stones	-	-	404,405	ebi-a-GCST90013889	European	34017140	2021

C-reactive protein	-	-	353,466	ebi-a-GCST90018950	European	34594039	2021

Note: AP=acute pancreatitis in European population; CP= chronic pancreatitis.

Supplement Table 2: MR estimates of GWAS summary data sets of European ancestry assessing the causal effect of glucocorticoid usage on pancreatitis, including sensitivity analyses of the MR findings

						Pleiotropy Test		Heterogeneity Test	
Exposure	Outcome	NSNP	Methods	OR (95% CI)	p value	MREgger Intercept (p Value)	MR-PRESSO (p Value)	Cochran's Q Test (p Value)	Rucker's Q Test (p Value)
glucocorticoid usage	AP	27	IVW	1.084 (0.945-1.242)	0.249	0.874	0.123	0.114	0.091
			Weighted Median	1.026 (0.863-1.219)	0.775				
			MR-Egger	1.049 (0.686-1.603)	0.828				
glucocorticoid usage	CP	27	IVW	1.027(0.850-1.240)	0.785	0.111	0.493	0.442	0.539
			Weighted Median	1.176 (0.909-1.523)	0.218				
			MR-Egger	1.625 (0.913-2.890)	0.111				

Note: AP, acute pancreatitis; CP, chronic pancreatitis.

Supplement Table 3: Multivariable Mendelian randomization analysis of glucocorticoid usage on the risk of pancreatitis using GWAS summary data sets of European ancestry.

OUTCOME	EXPOSURE	N SNP	BETA	SE	OR	95%LCI	95%UCI	P-value
CP	Cholelithiasis	20	0.19012	0.05869	1.20939	1.07798	1.35683	0.0012
CP	C-reactive protein	110	0.00799	0.10286	1.00802	0.82397	1.23318	0.93811
CP	Glucocorticoids	10	0.16206	0.10265	1.17593	0.96162	1.438	0.1144
CP	Body mass index	344	-0.11054	0.14034	0.89535	0.68004	1.17884	0.43091
CP	Alcohol intake frequency	31	-0.14516	0.18183	0.86489	0.60559	1.23521	0.4247
AP	Cholelithiasis	20	0.42211	0.03713	1.52517	1.41812	1.6403	6.02E-30
AP	C-reactive protein	110	0.06334	0.06347	1.06538	0.94076	1.20652	0.31834
AP	Glucocorticoids	10	0.07112	0.06351	1.07371	0.94805	1.21604	0.26275
AP	Body mass index	344	-0.00637	0.0881	0.99365	0.83606	1.18094	0.94236
AP	Alcohol intake frequency	31	-0.05571	0.11809	0.94581	0.75039	1.19212	0.63709

Supplement Table 4: MR estimates of GWAS summary data sets of FinnGen Biobank Consortium assessing the causal effect of glucocorticoid usage on pancreatitis, including sensitivity analyses of the MR findings

Exposure	Outcome	NSNP	Methods	OR (95% CI)	p value	Pleiotropy Test		Heterogeneity Test	
						MREgger Intercept (p Value)	MR-PRESSO (p Value)	Cochran's Q Test (p Value)	Rucker's Q Test (p Value)
glucocorticoid usage	AP	26	IVW	1.130 (0.921-1.386)	0.243	0.920	<0.001 (one outlier SNP)	0.0019	0.0012
			Weighted Median	0.963 (0.771-1.202)	0.737				
			MR-Egger	1.094 (0.567-2.110)	0.791				
glucocorticoid usage	CP	26	IVW	0.982(0.798-1.209)	0.864	0.243	0.269	0.217	0.239
			Weighted Median	0.996 (0.751-1.322)	0.979				
			MR-Egger	1.429 (0.748-2.730)	0.291				

Supplement Table 5: MR estimates of GWAS summary data sets of East Asian descendants assessing the causal effect of glucocorticoid usage on pancreatitis, including sensitivity analyses of the MR findings

Exposure	Outcome	NSNP	Methods	OR (95% CI)	p value	Pleiotropy Test		Heterogeneity Test	
						MREgger Intercept (p Value)	MR-PRESSO (p Value)	Cochran's Q Test (p Value)	Rucker's Q Test (p Value)
glucocorticoid usage	AP	55	IVW	0.859 (0.682-1.083)	0.199	0.846	0.602	0.551	0.513
			Weighted Median	0.962 (0.698-1.325)	0.812				
			MR-Egger	0.796 (0.357-1.775)	0.580				
glucocorticoid usage	CP	55	IVW	1.038(0.761-1.415)	0.816	0.392	0.576	0.537	0.527
			Weighted Median	1.064 (0.674-1.679)	0.791				
			MR-Egger	0.660 (0.223-1.930)	0.452				

Supplement Table 6: Multivariable Mendelian randomization analysis of glucocorticoid usage on the risk of pancreatitis using GWAS summary data sets of FinnGen Biobank Consortium.

OUTCOME	EXPOSURE	N SNP	BETA	SE	OR	95%LCI	95%UCI	P-value
CP	Cholelithiasis	20	0.1855	0.05957	1.20382	1.07116	1.35291	0.00185
CP	C-reactive protein	111	-0.00117	0.09971	0.99883	0.82153	1.21441	0.99066
CP	Glucocorticoids	10	0.08636	0.10652	1.0902	0.88477	1.34332	0.41755
CP	Body mass index	338	0.05042	0.15482	1.05171	0.77644	1.42456	0.7447
CP	Alcohol intake frequency	31	-0.11349	0.24404	0.89271	0.55332	1.44026	0.64189
AP	Cholelithiasis	20	0.32118	0.04473	1.37876	1.26303	1.50508	6.92E-13
AP	C-reactive protein	111	0.01173	0.07432	1.0118	0.87465	1.17046	0.87457
AP	Glucocorticoids	10	0.06275	0.07935	1.06476	0.9114	1.24393	0.42906
AP	Body mass index	338	0.07136	0.11532	1.07397	0.8567	1.34633	0.53605
AP	Alcohol intake frequency	31	-0.13745	0.18174	0.87158	0.61039	1.24454	0.44948

2 Supplementary Figures

Supplementary Figure 1 Funnel plot on glucocorticoid usage and AP, which visualize overall heterogeneity of Mendelian randomization (MR) effect estimates using GWAS summary data sets of European ancestry.

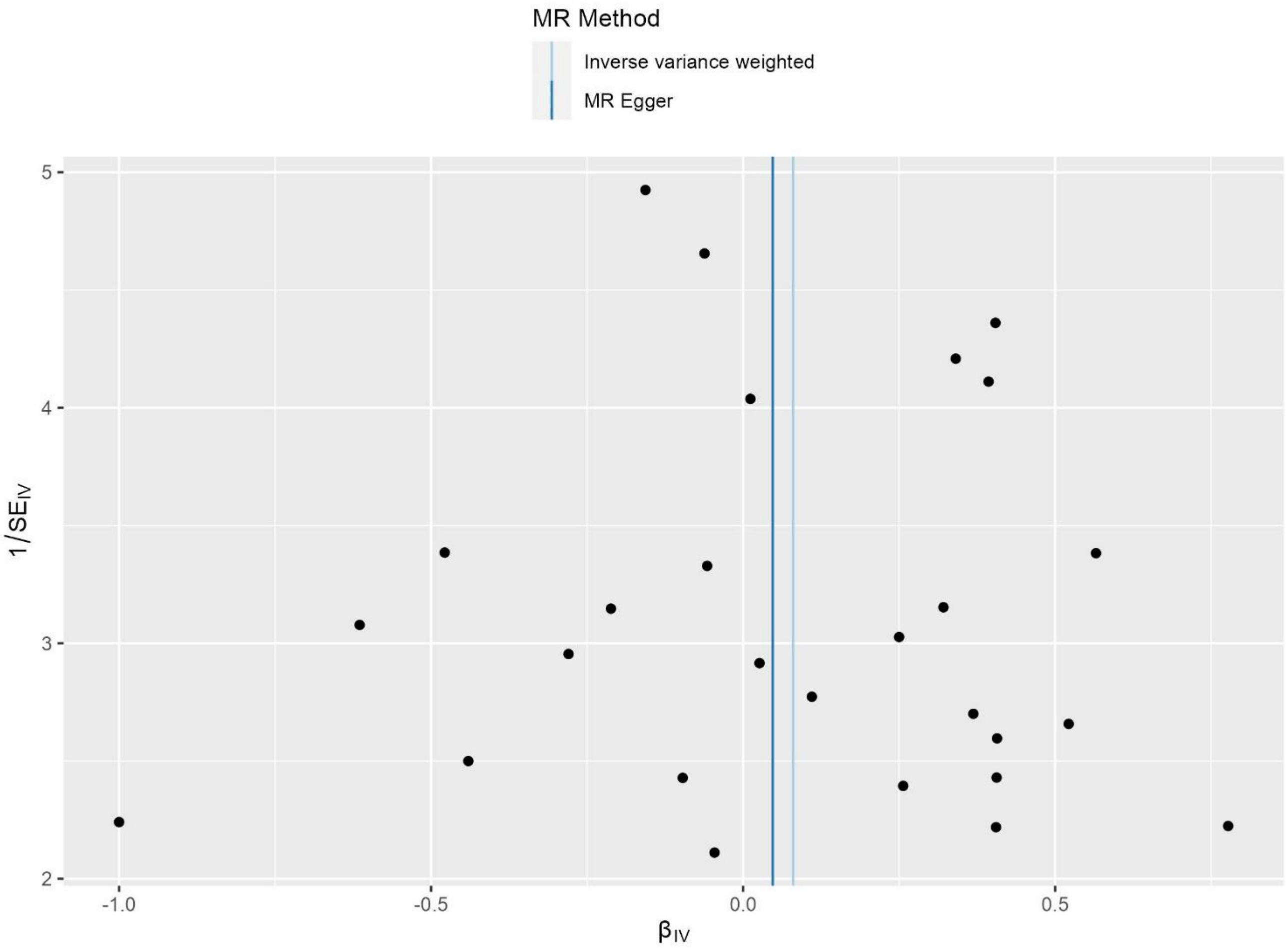

Supplementary Figure 2 Funnel plot on glucocorticoid usage and CP which visualize overall heterogeneity of Mendelian randomization (MR) effect estimates using GWAS summary data sets of European ancestry.

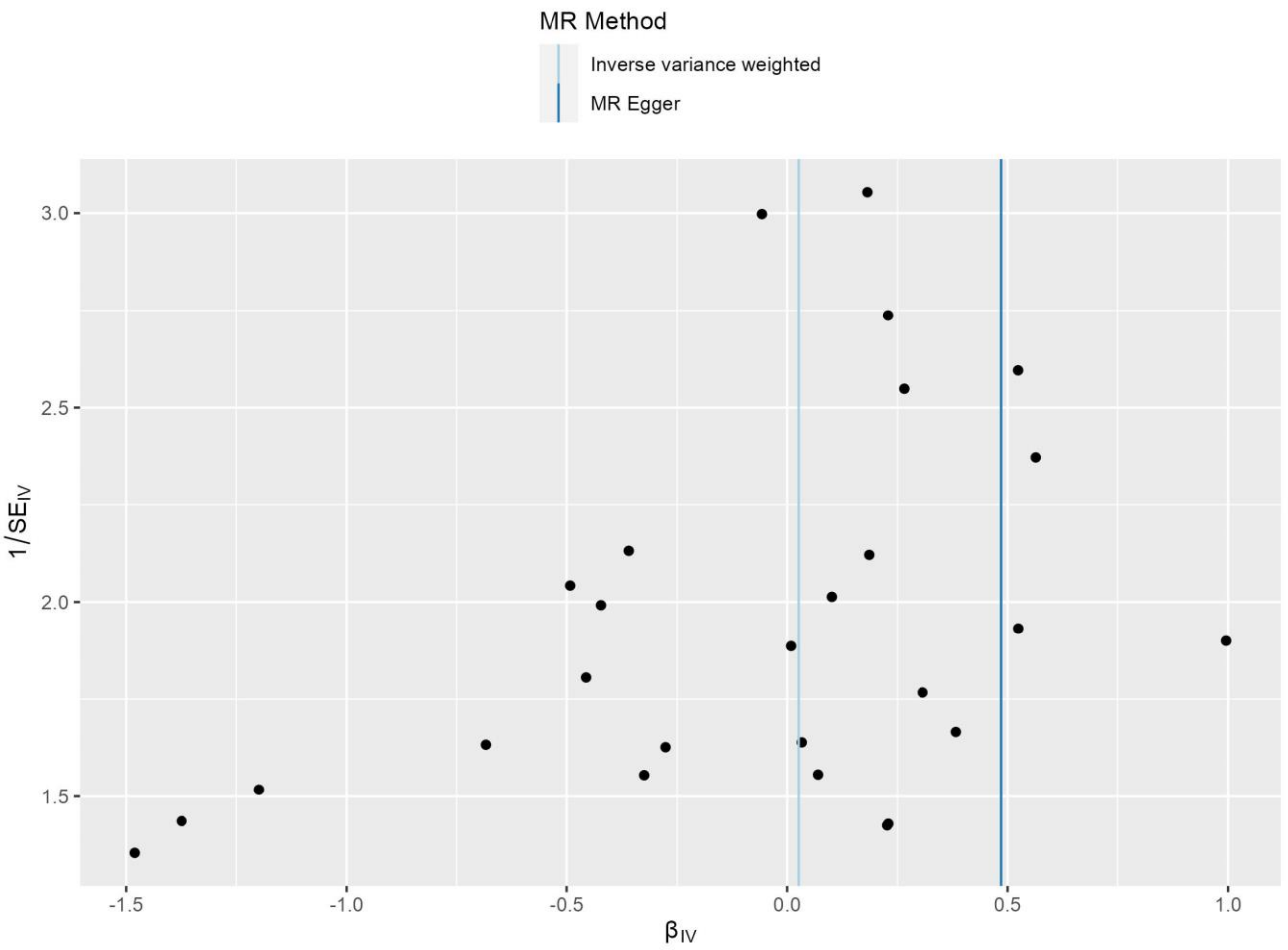

Supplementary Figure 3 Leave-one-out plot of glucocorticoid usage and AP using GWAS summary data sets of European ancestry. It illustrates how the exclusion of each SNP affects the causal estimations (point with horizontal line) for the effect of glucocorticoid usage on AP.

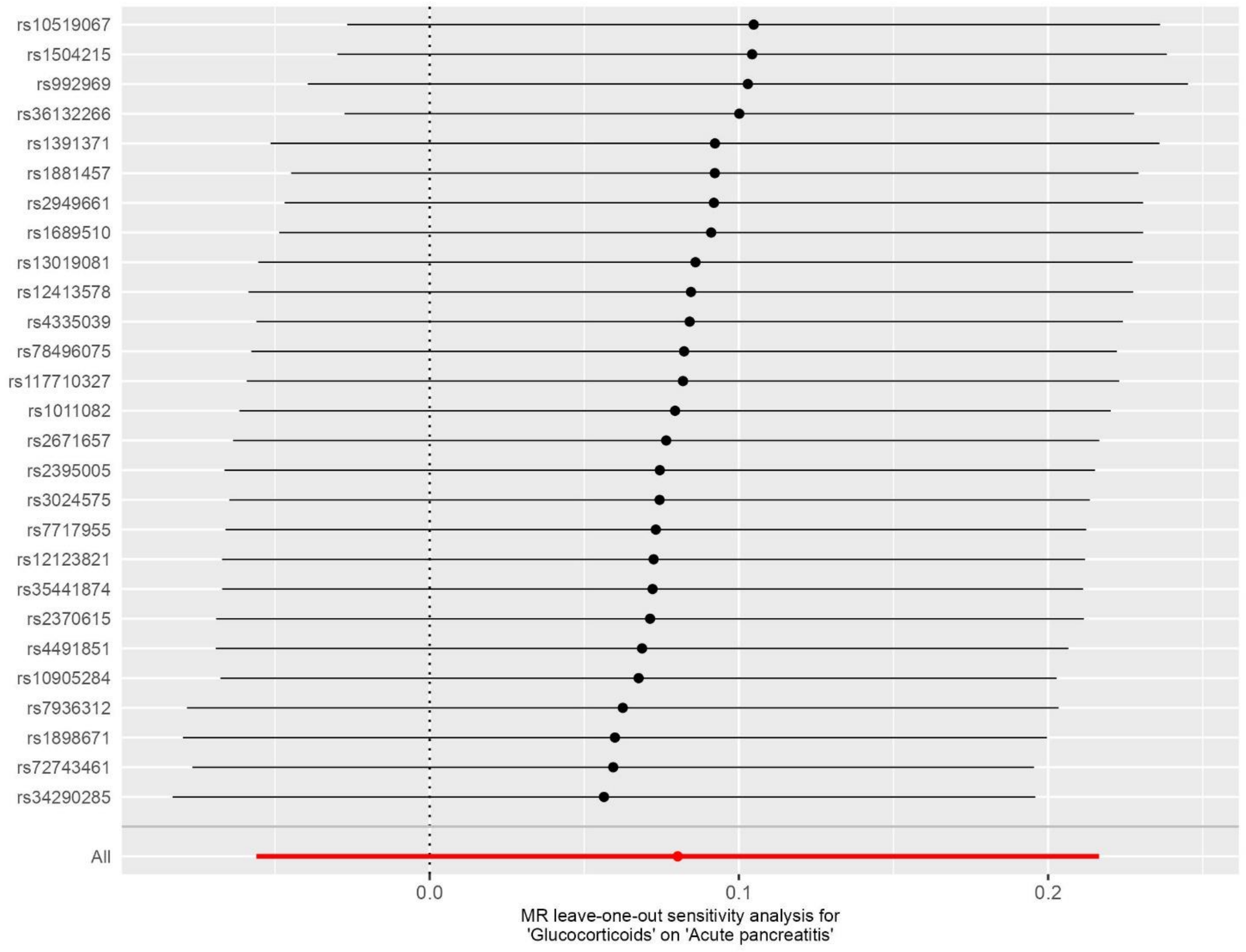

Supplementary Figure 4 Leave-one-out plot of glucocorticoid usage and CP using GWAS summary data sets of European ancestry. It illustrates how the exclusion of each SNP affects the causal estimations (point with horizontal line) for the effect of glucocorticoid usage on CP.

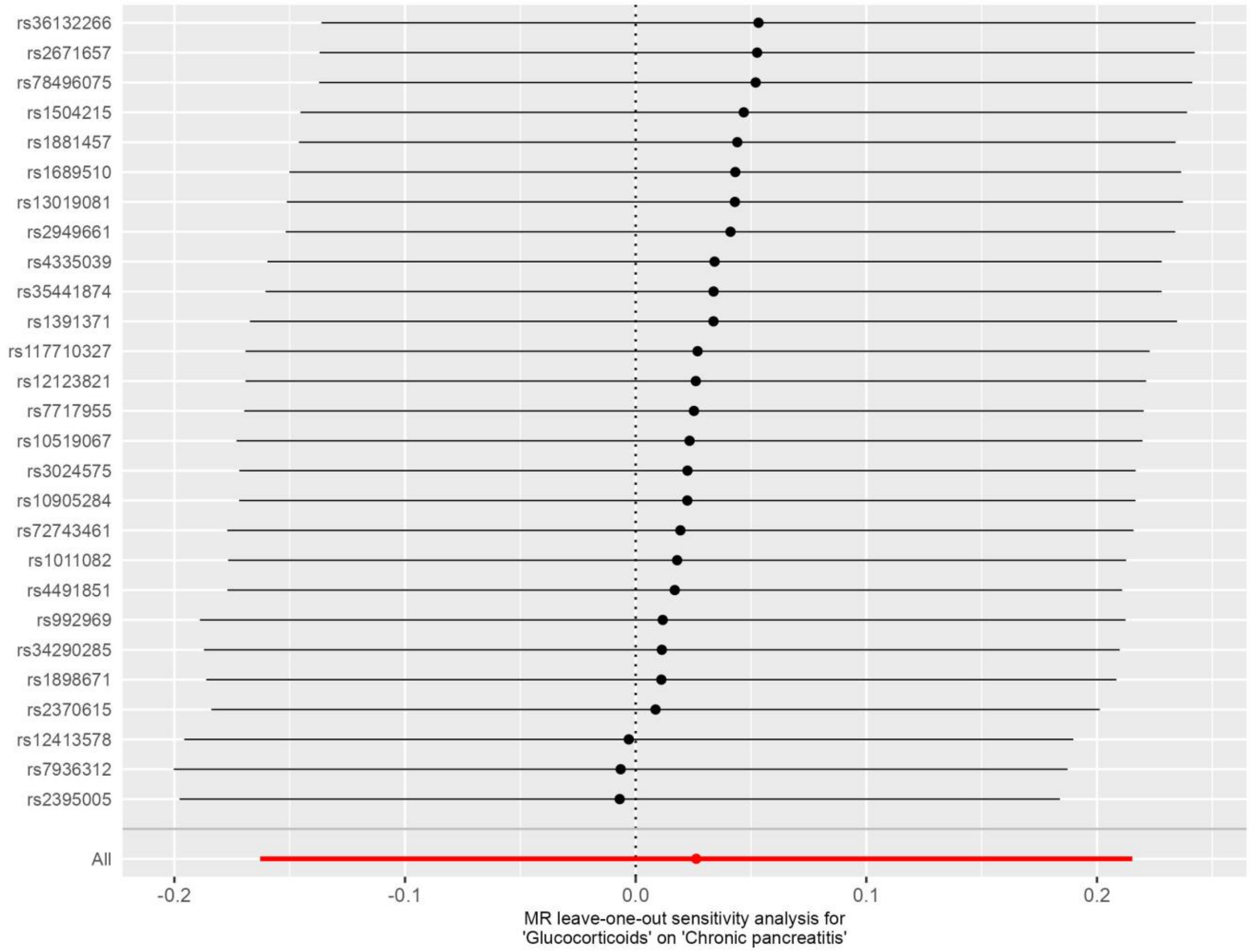

Supplementary Figure 5 The scatter plot illustrates the causal effect of glucocorticoid usage on the risk of acute pancreatitis (AP) using GWAS summary data sets of FinnGen Biobank Consortium. The slope of the line indicates the strength of this causal relationship. MR denotes Mendelian randomization.

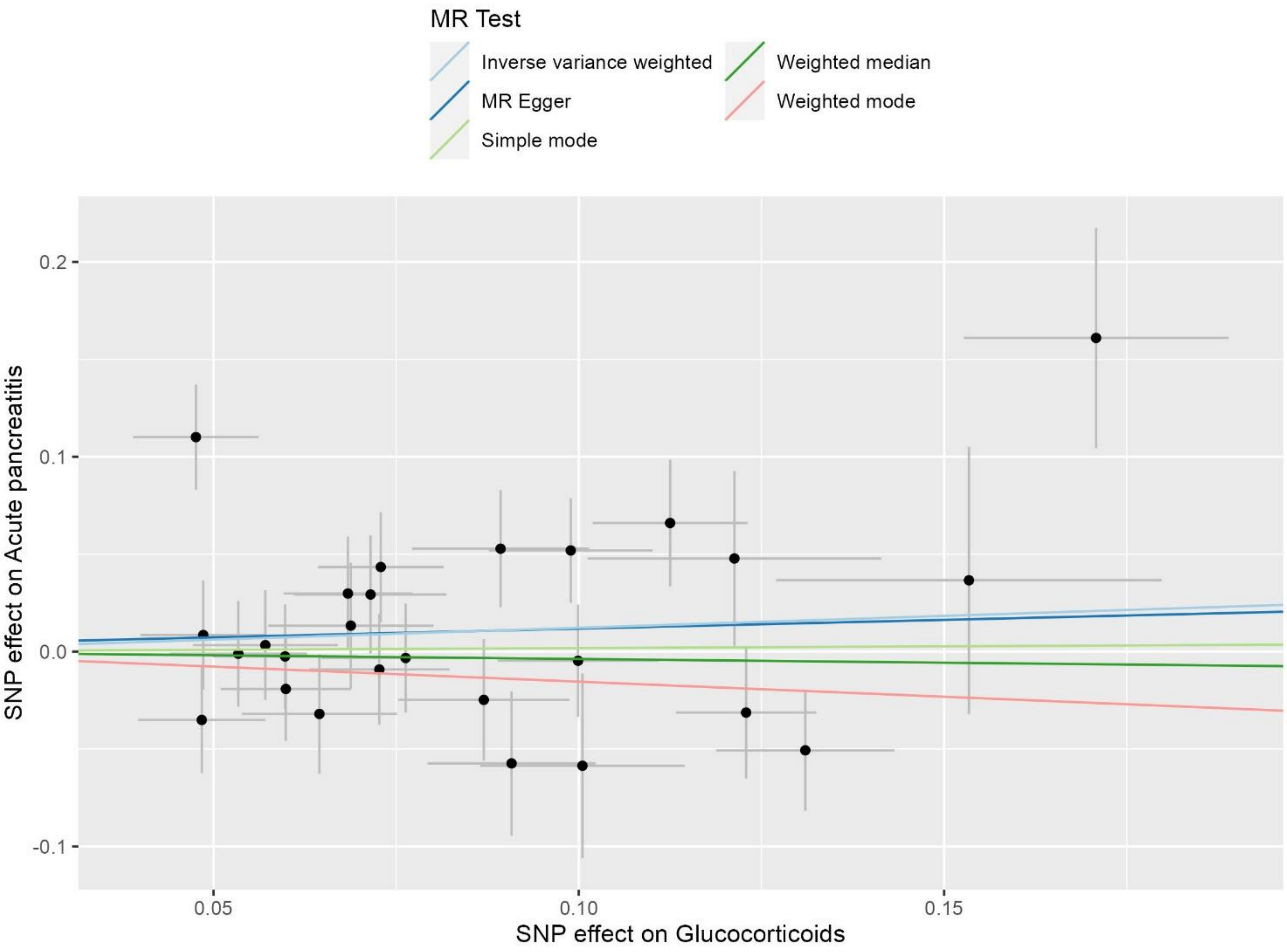

Supplementary Figure 6 Forest plots illustrating the causal relationship between individual SNPs and the risk of acute pancreatitis (AP) using GWAS summary data sets of FinnGen Biobank Consortium.

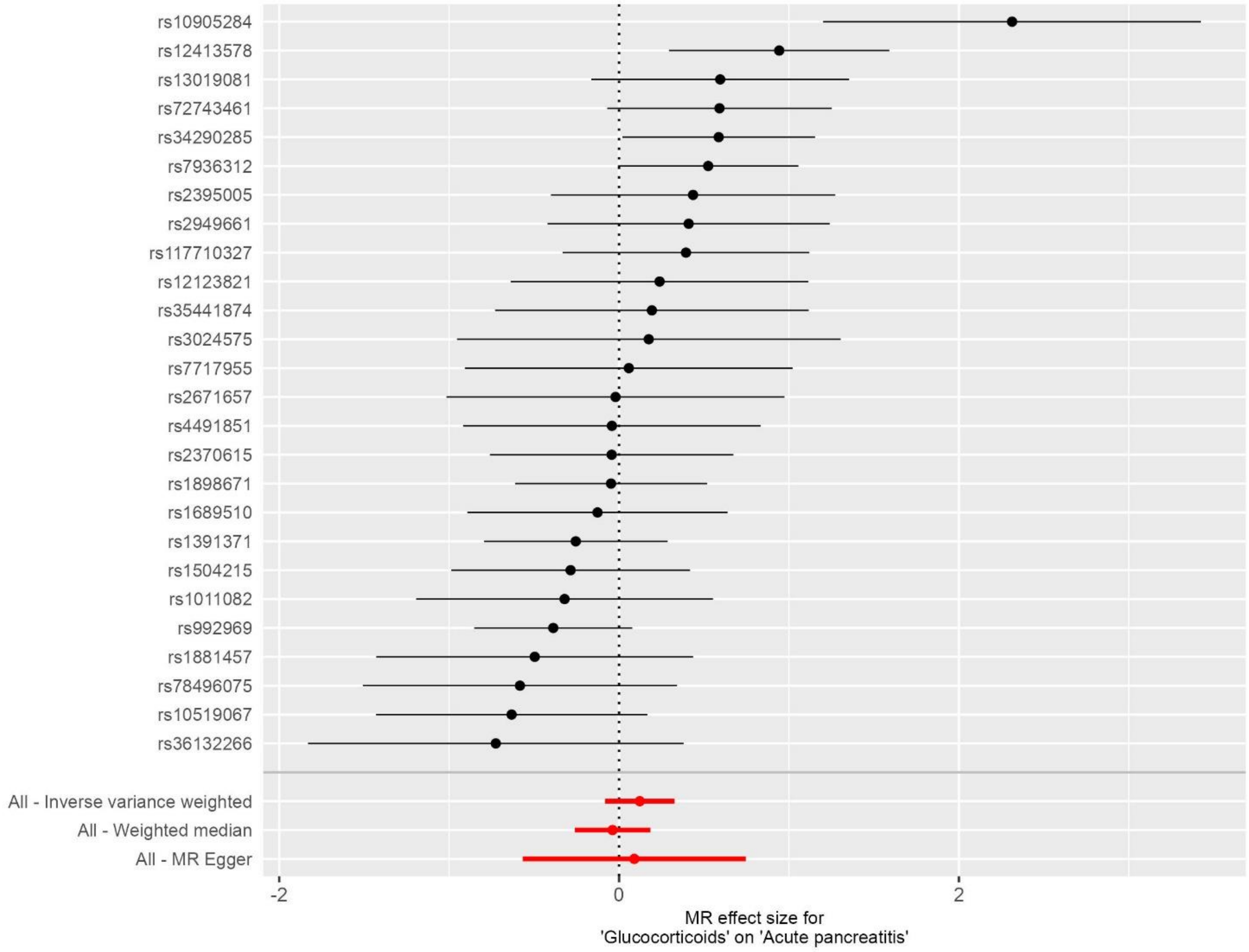

Supplementary Figure 7 The scatter plot illustrates the causal effect of glucocorticoid usage on the risk of acute pancreatitis (AP) using GWAS summary data sets of East Asian descendants. The slope of the line indicates the strength of this causal relationship. MR denotes Mendelian randomization.

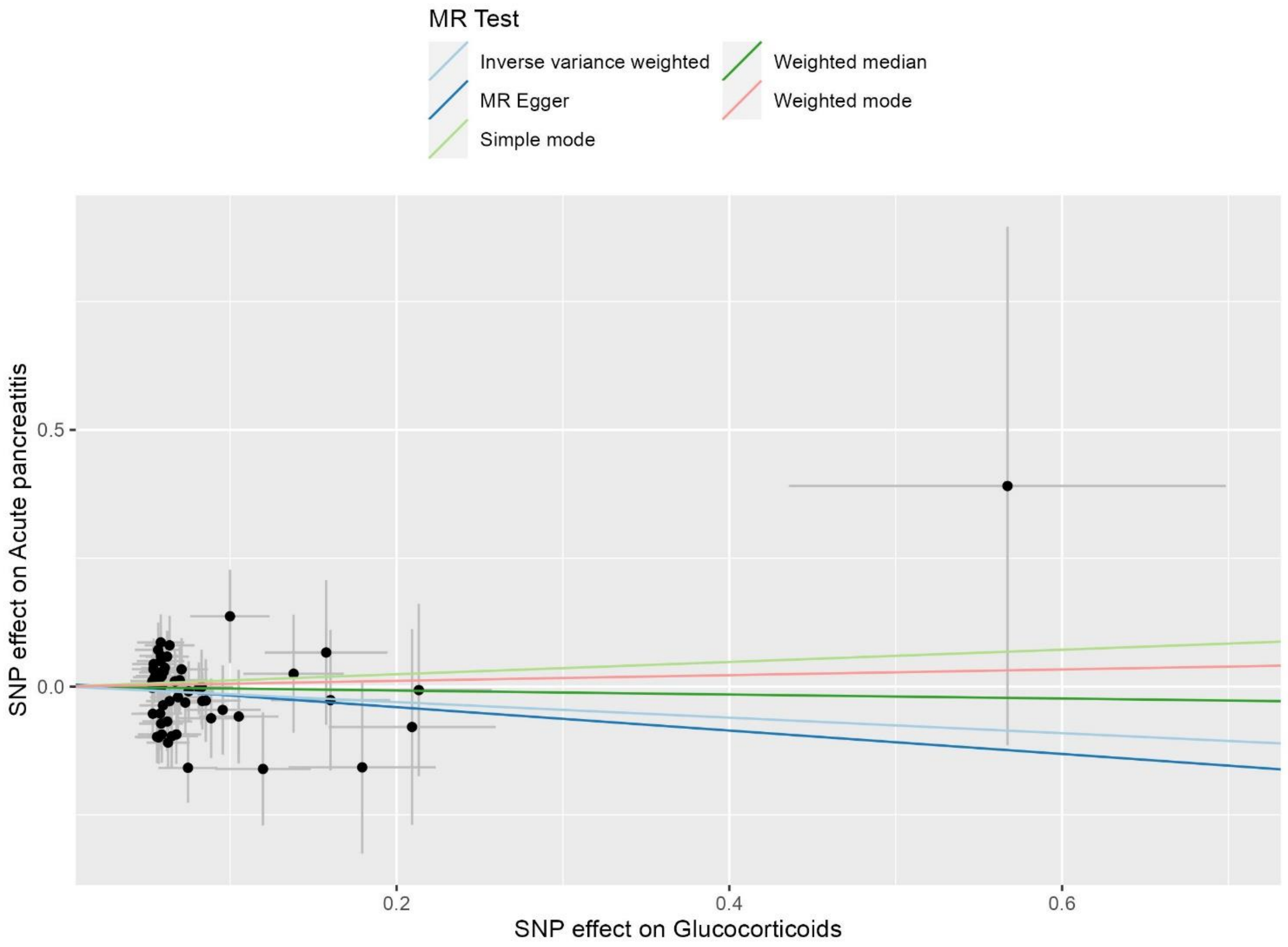

Supplementary Figure 8 Forest plots illustrating the causal relationship between individual SNPs and the risk of acute pancreatitis (AP) using GWAS summary data sets of East Asian descendants.

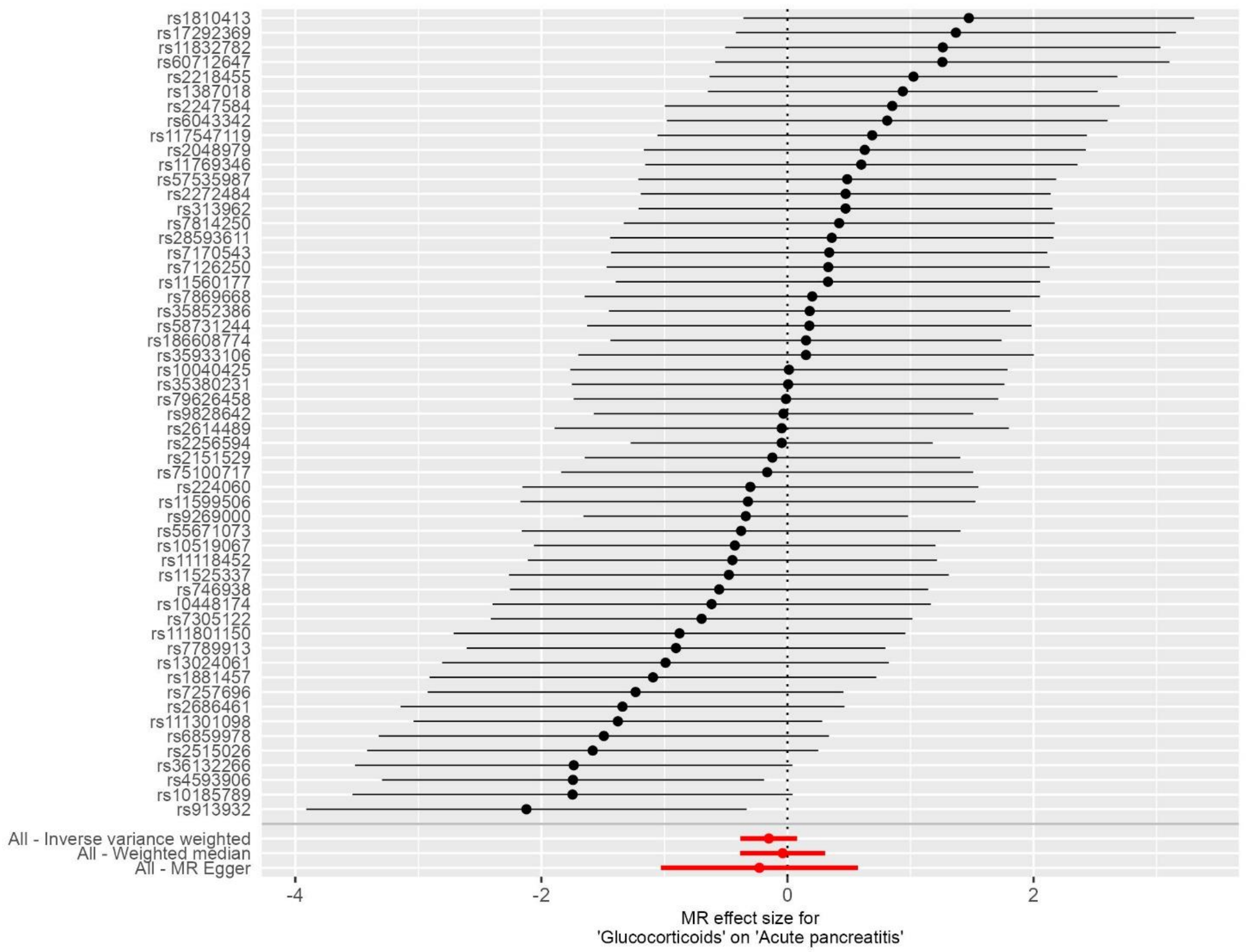

Supplementary Figure 9 The scatter plot illustrates the causal effect of glucocorticoid usage on the risk of chronic pancreatitis (CP) using GWAS summary data sets of FinnGen Biobank Consortium. The slope of the line indicates the strength of this causal relationship. MR denotes Mendelian randomization.

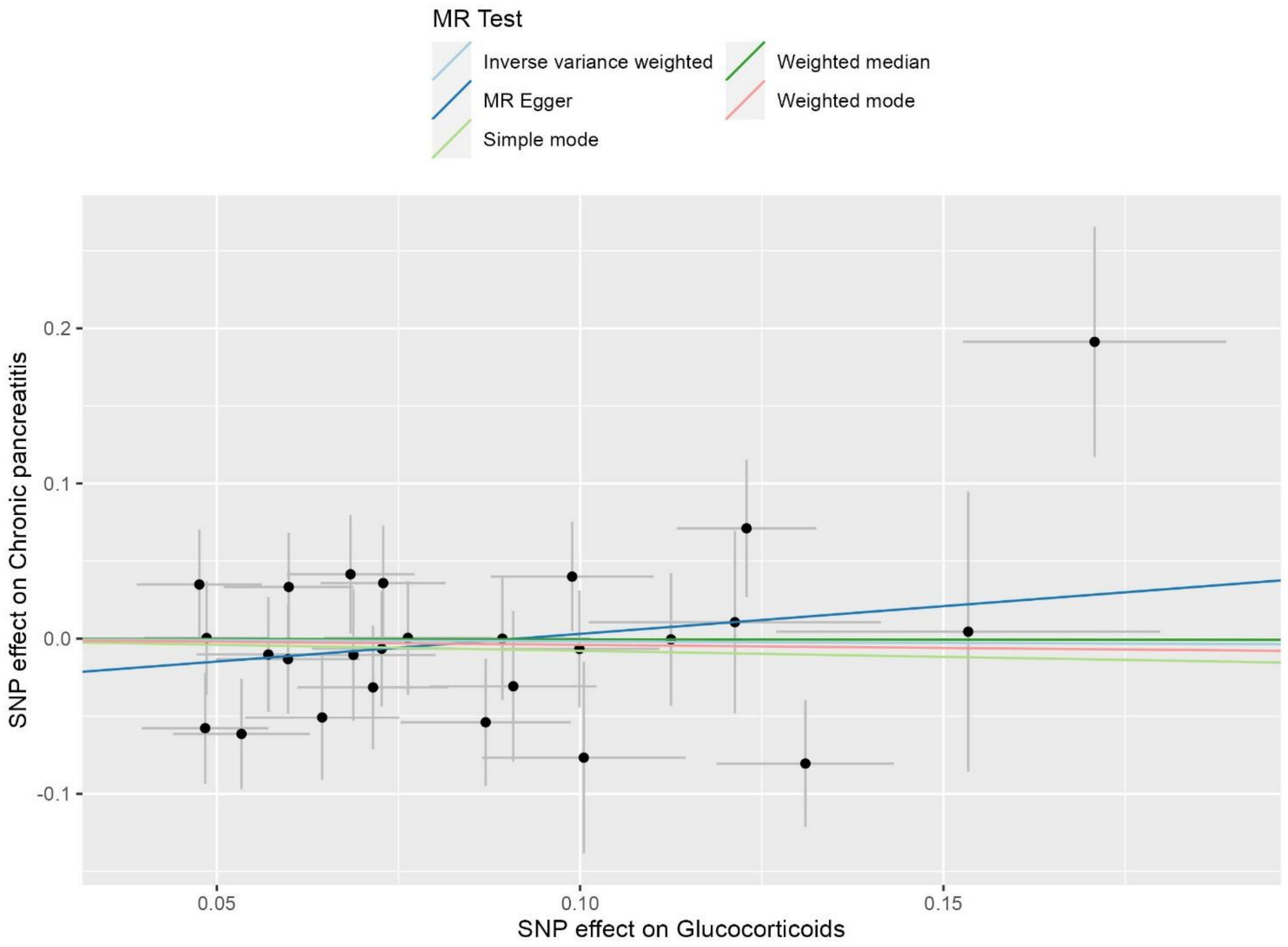

Supplementary Figure 10 Forest plots illustrating the causal relationship between individual SNPs and the risk of chronic pancreatitis (CP) using GWAS summary data sets of FinnGen Biobank Consortium.

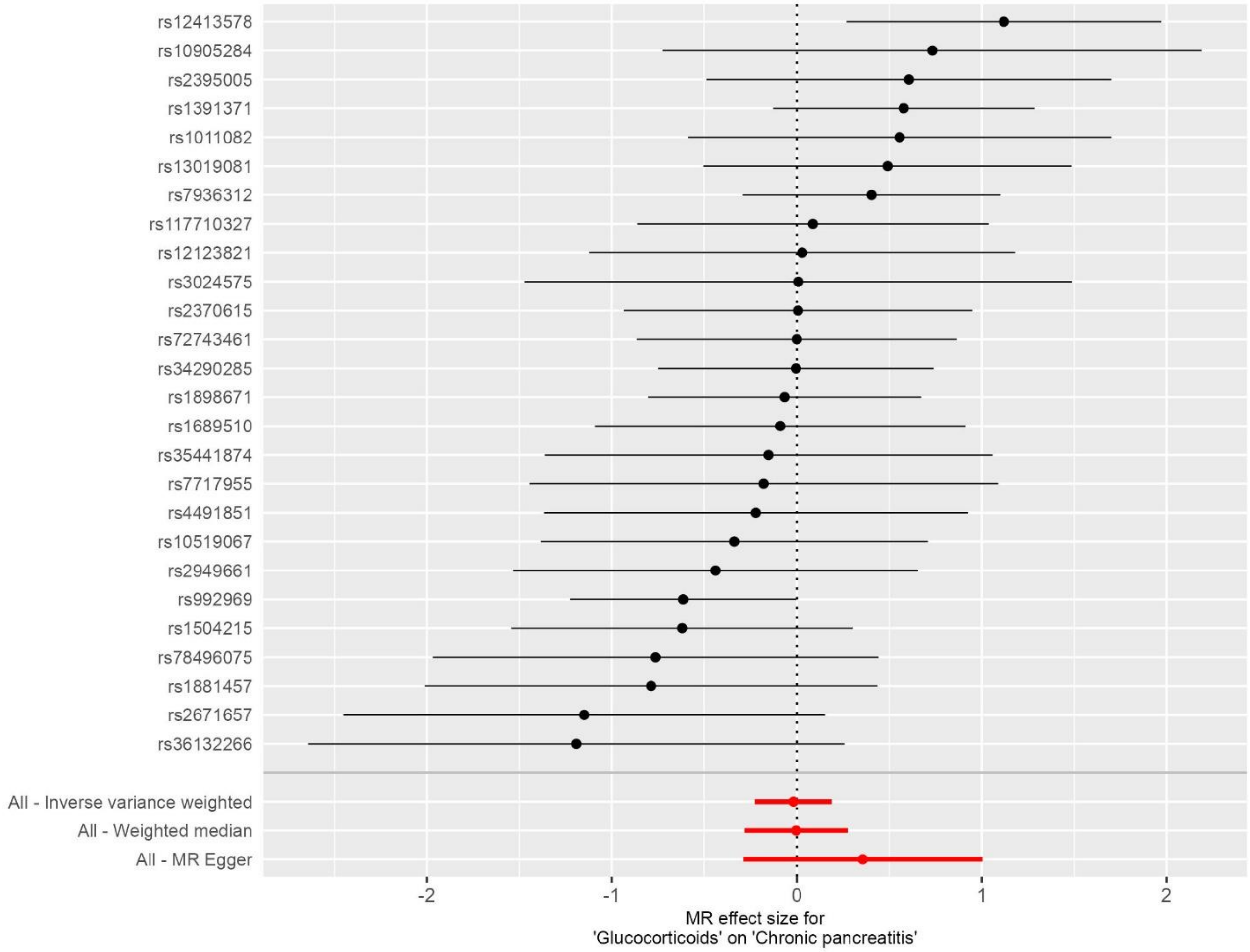

Supplementary Figure 11 The scatter plot illustrates the causal effect of glucocorticoid usage on the risk of chronic pancreatitis (CP) using GWAS summary data sets of East Asian descendants. The slope of the line indicates the strength of this causal relationship. MR denotes Mendelian randomization.

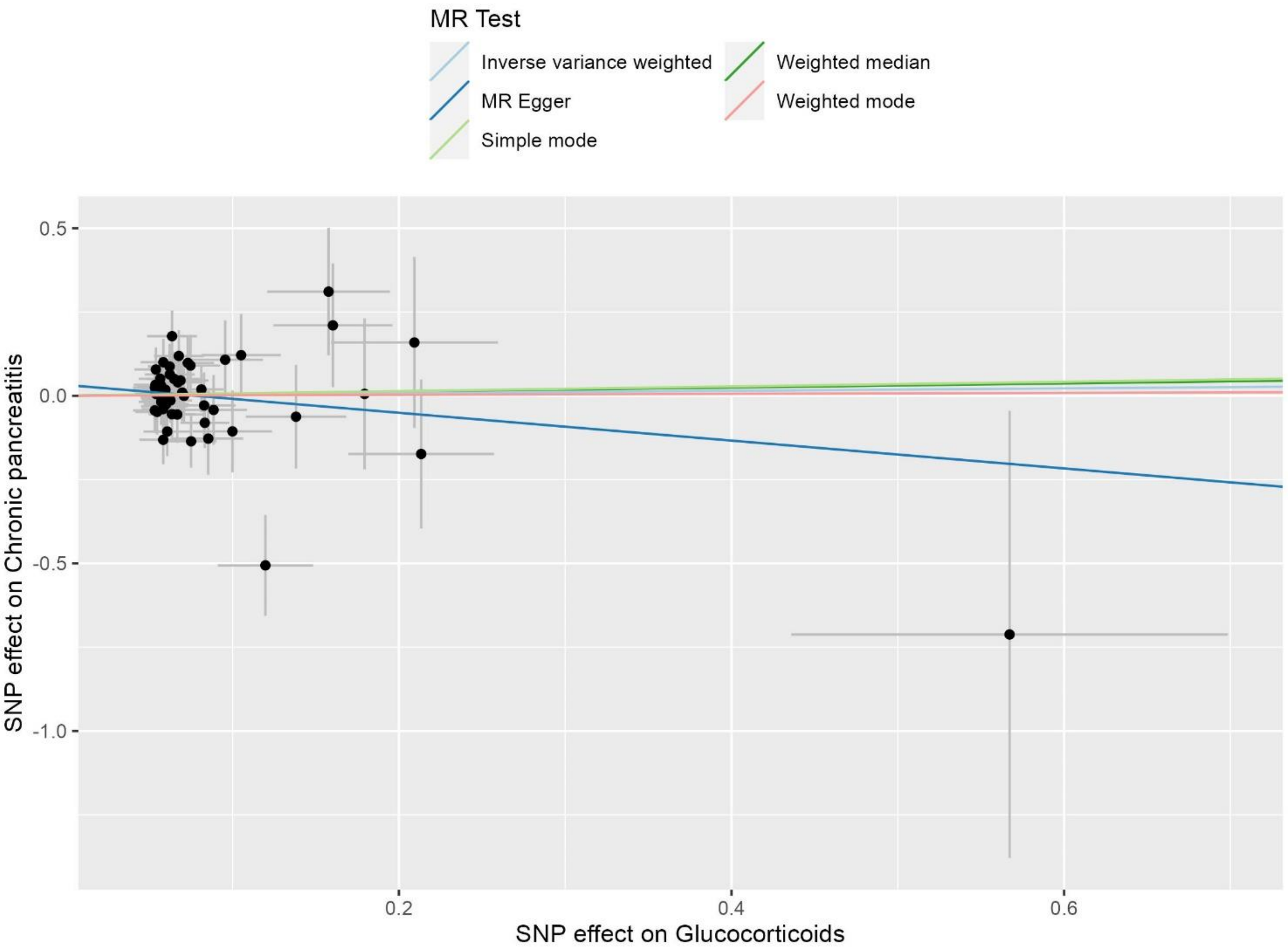

Supplementary Figure 12 Forest plots illustrating the causal relationship between individual SNPs and the risk of chronic pancreatitis (CP) using GWAS summary data sets of East Asian descendants..

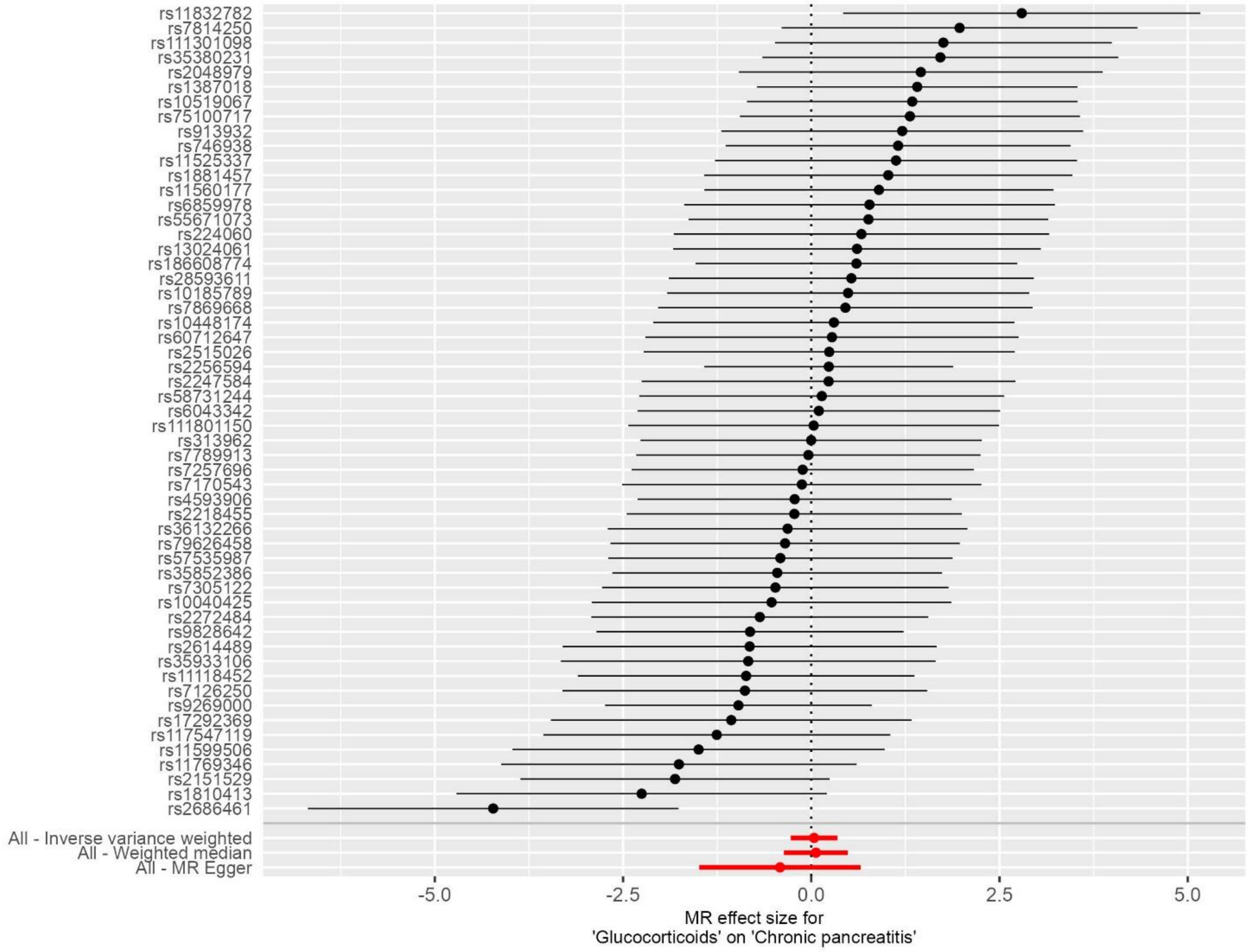

Supplementary Figure 13 Funnel plot on glucocorticoid usage and AP, which visualize overall heterogeneity of Mendelian randomization (MR) effect estimates using GWAS summary data sets of FinnGen Biobank Consortium.

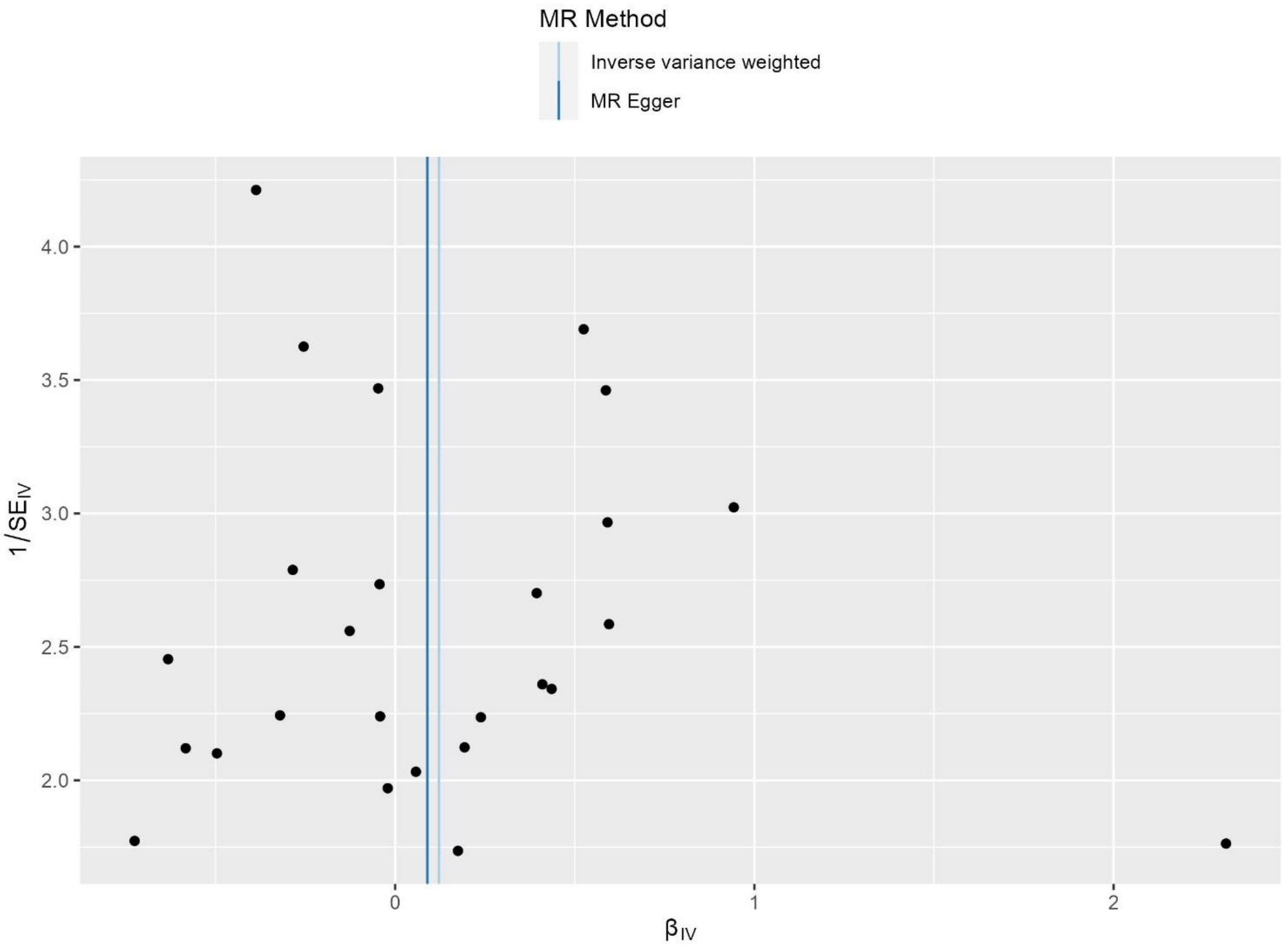

Supplementary Figure 14 Funnel plot on glucocorticoid usage and CP which visualize overall heterogeneity of Mendelian randomization (MR) effect estimates using GWAS summary data sets of FinnGen Biobank Consortium.

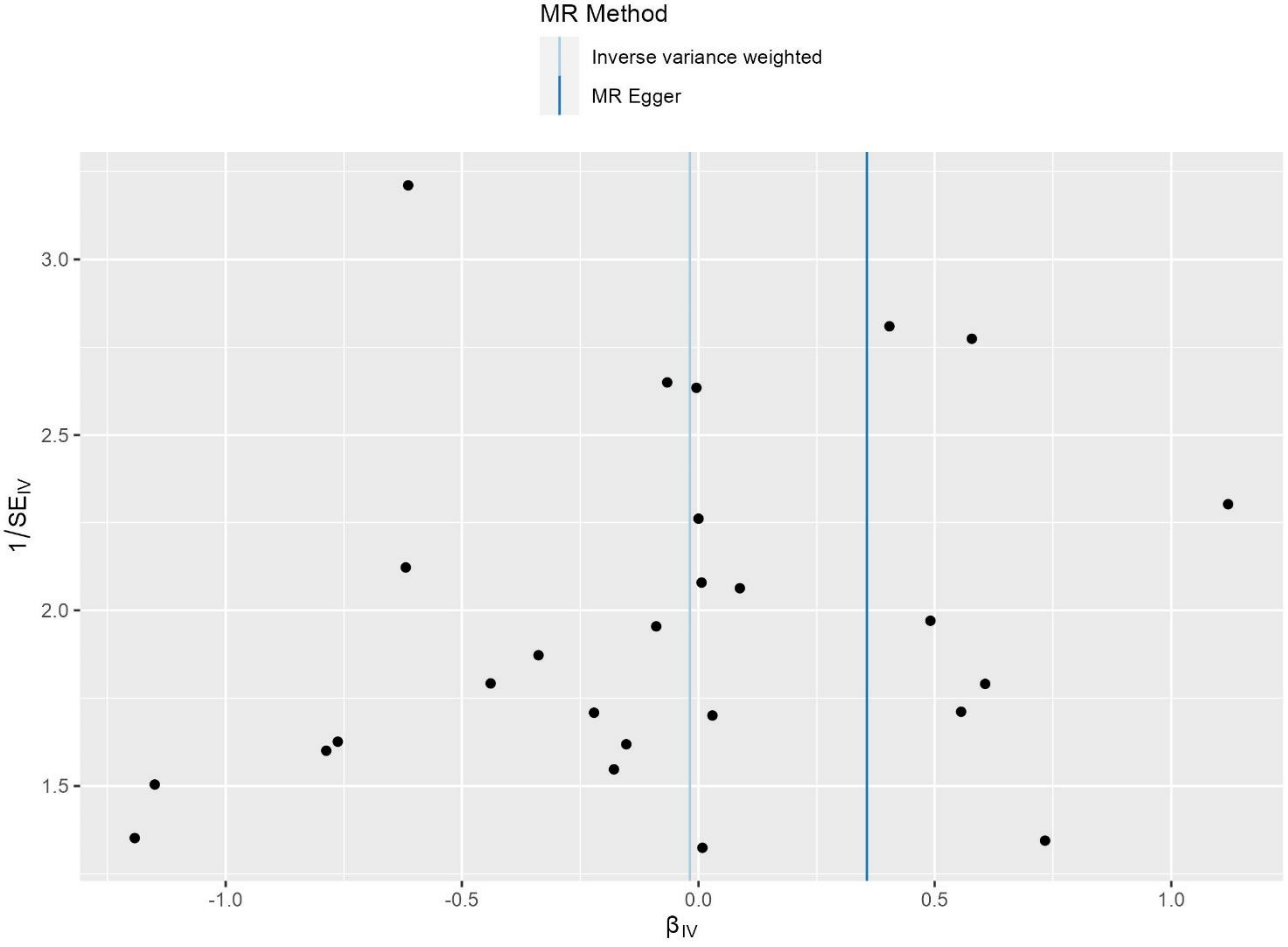

Supplementary Figure 15 Leave-one-out plot of glucocorticoid usage and AP using GWAS summary data sets of FinnGen Biobank Consortium. It illustrates how the exclusion of each SNP affects the causal estimations (point with horizontal line) for the effect of glucocorticoid usage on AP.

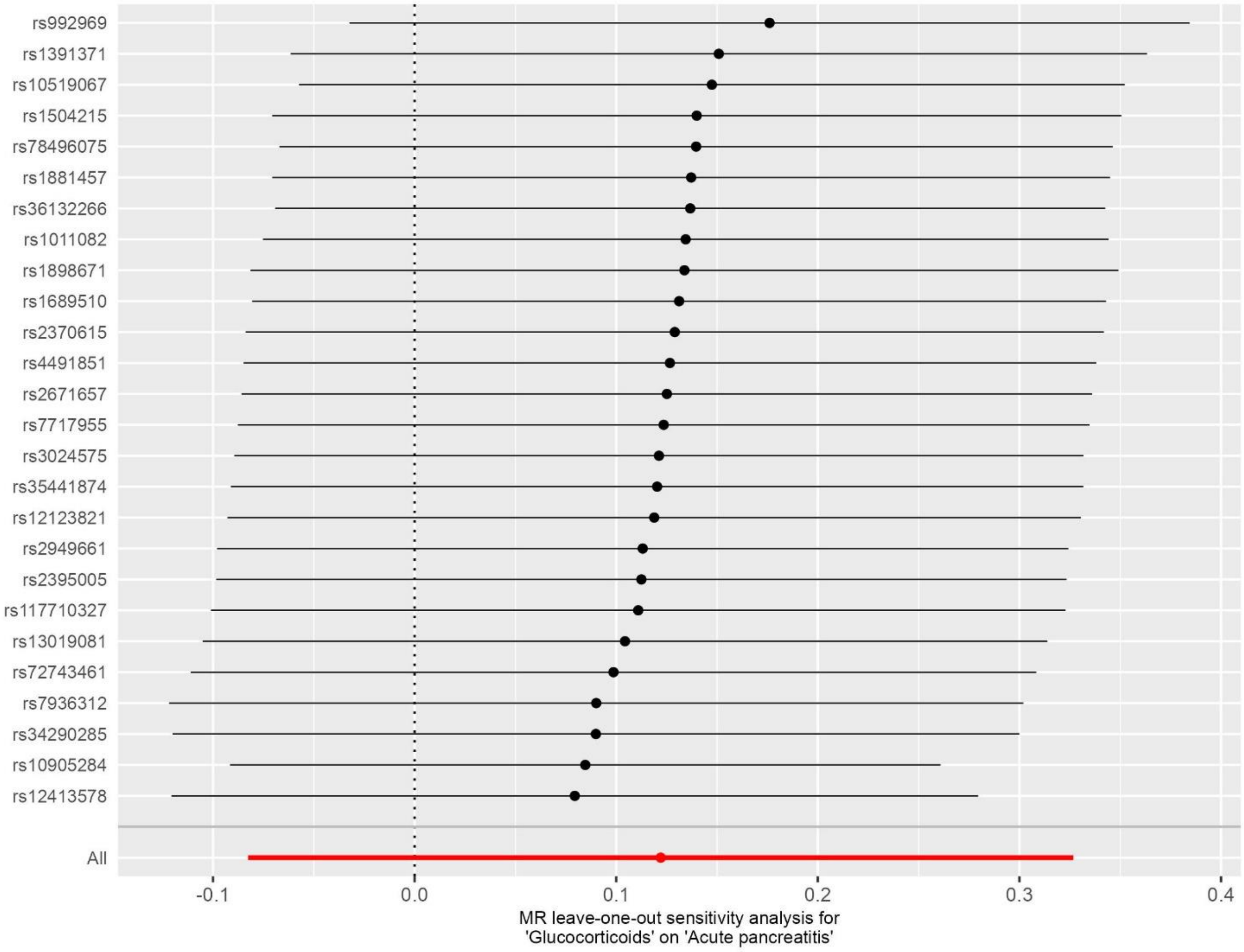

Supplementary Figure 16 Leave-one-out plot of glucocorticoid usage and CP using GWAS summary data sets of FinnGen Biobank Consortium. It illustrates how the exclusion of each SNP affects the causal estimations (point with horizontal line) for the effect of glucocorticoid usage on CP.

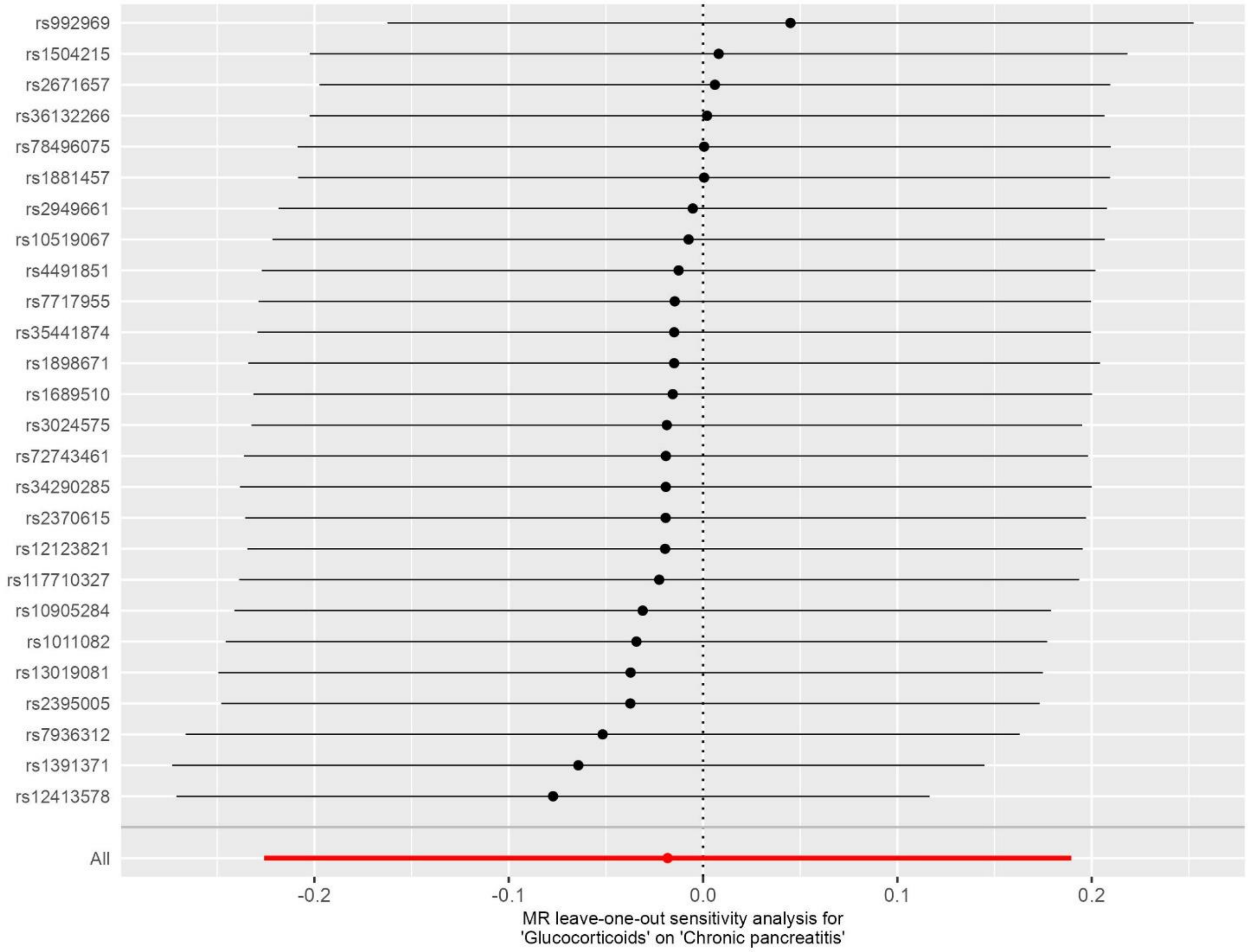

Supplementary Figure 17 Funnel plot on glucocorticoid usage and AP, which visualize overall heterogeneity of Mendelian randomization (MR) effect estimates using GWAS summary data sets of East Asian descendants.

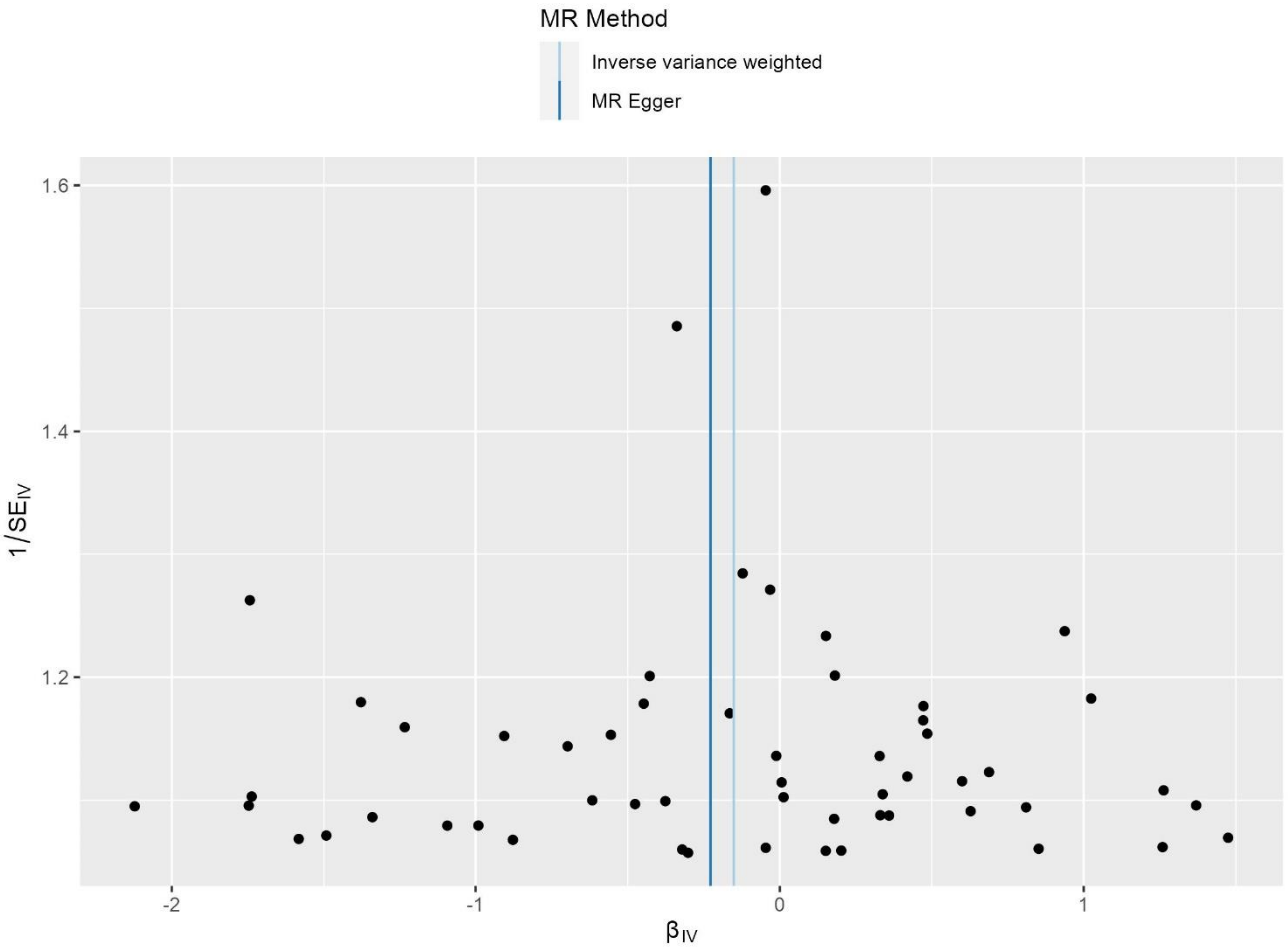

Supplementary Figure 18 Funnel plot on glucocorticoid usage and CP which visualize overall heterogeneity of Mendelian randomization (MR) effect estimates using GWAS summary data sets of East Asian descendants.

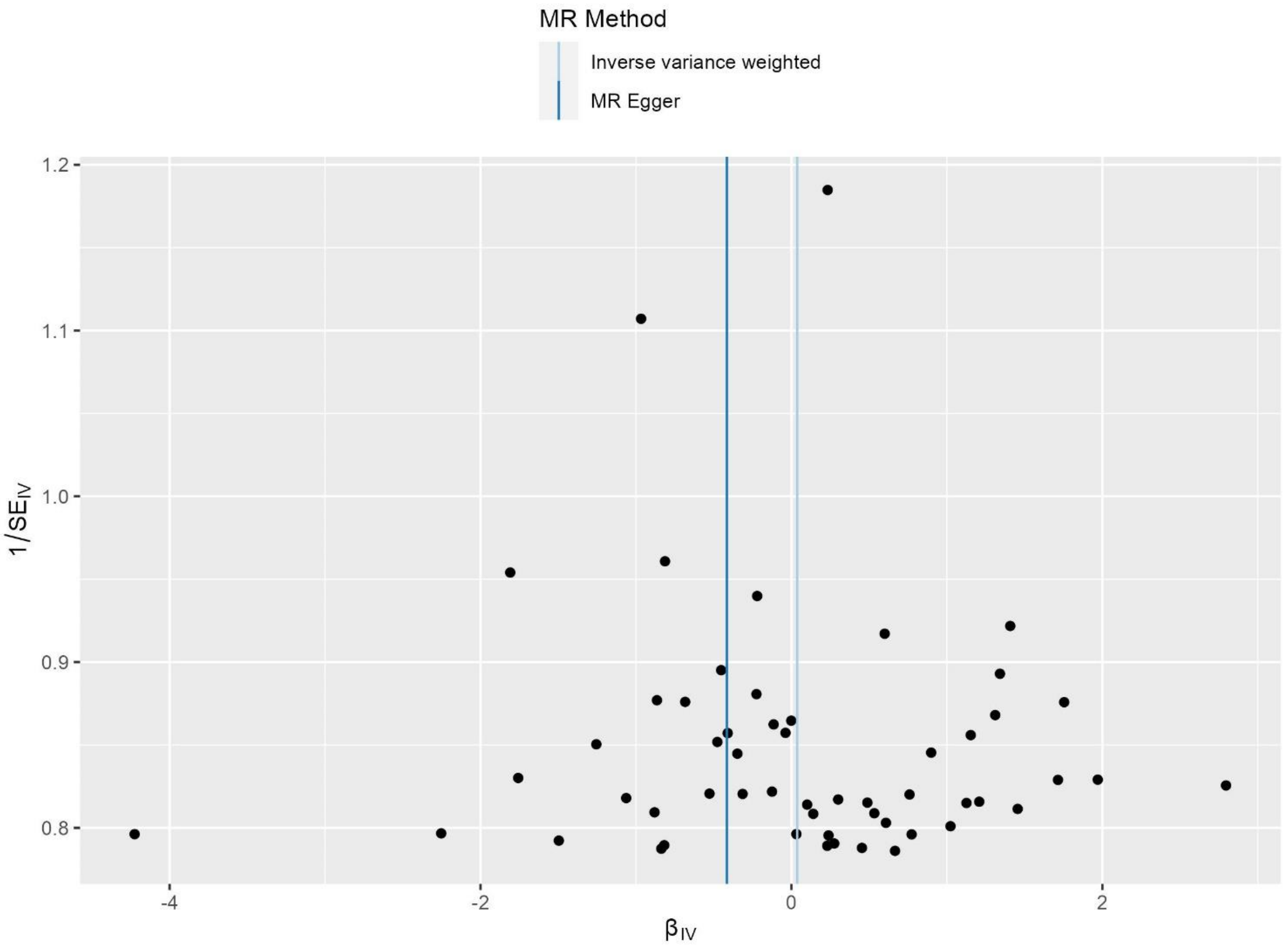

Supplementary Figure 19 Leave-one-out plot of glucocorticoid usage and AP using GWAS summary data sets of East Asian descendants. It illustrates how the exclusion of each SNP affects the causal estimations (point with horizontal line) for the effect of glucocorticoid usage on AP.

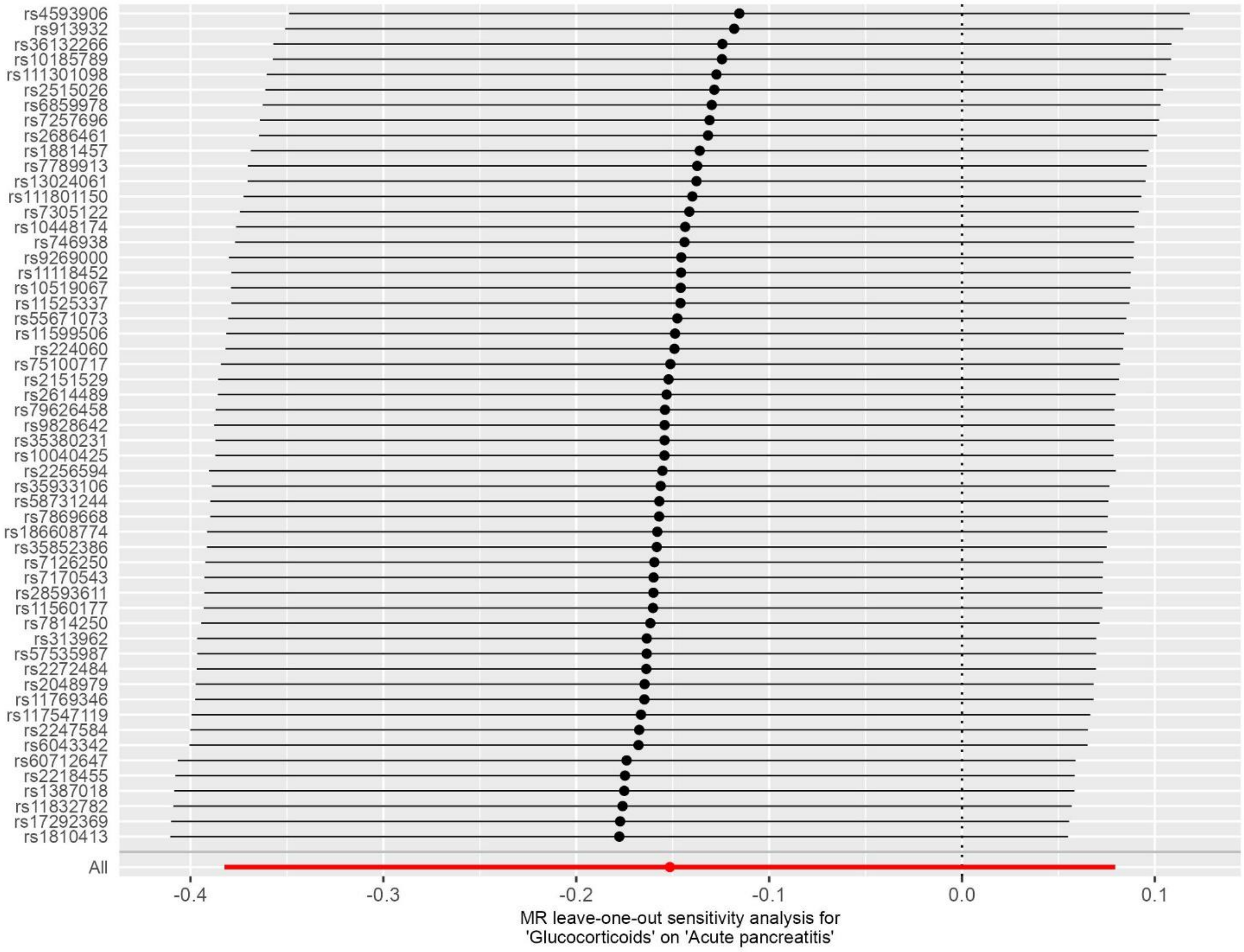

Supplementary Figure 20 Leave-one-out plot of glucocorticoid usage and CP using GWAS summary data sets of East Asian descendants. It illustrates how the exclusion of each SNP affects the causal estimations (point with horizontal line) for the effect of glucocorticoid usage on CP.

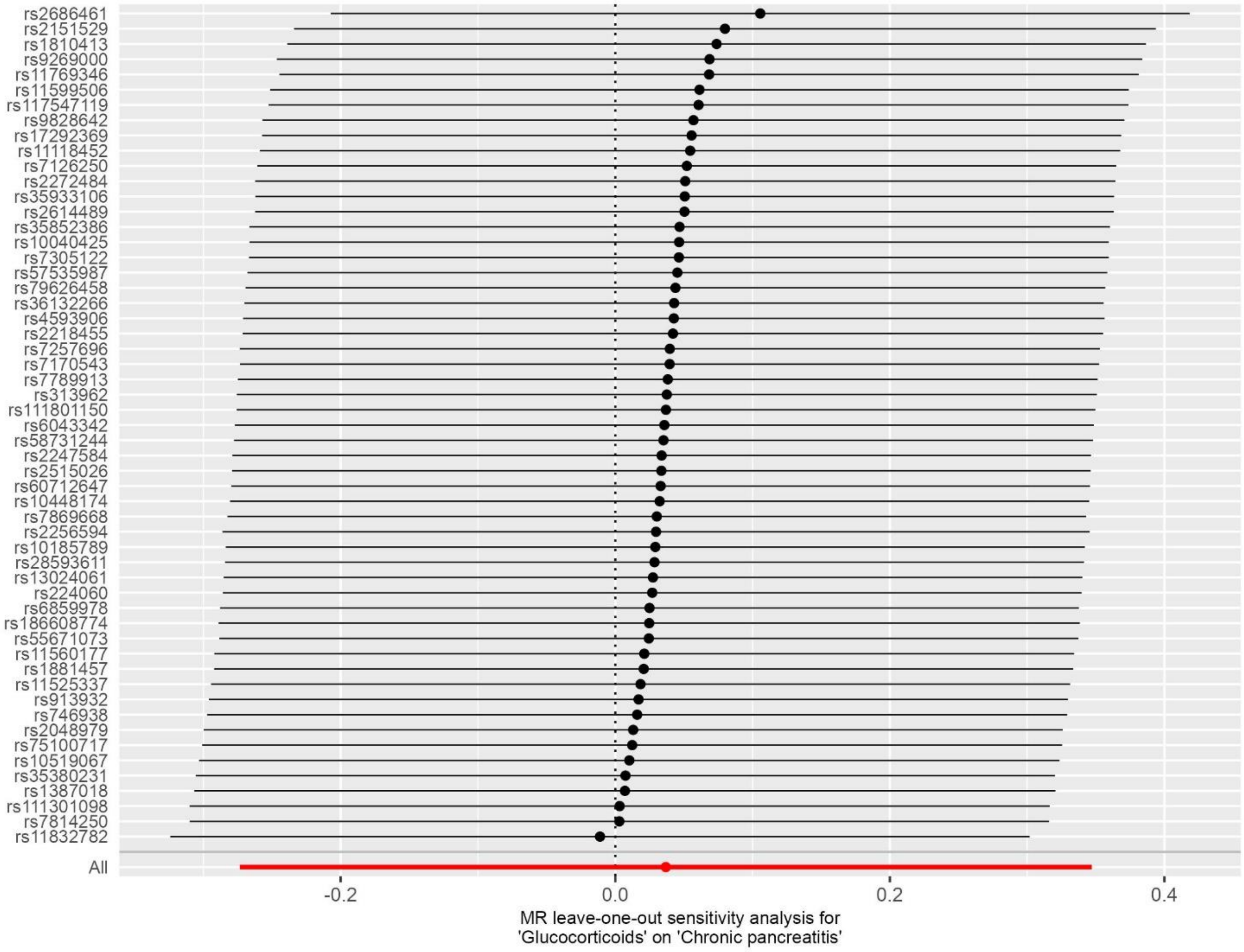

Supplementary Figure 21 Multivariable Mendelian randomization of glucocorticoid usage on the risk of pancreatitis sing GWAS summary data sets of FinnGen Biobank Consortium. Error bars represent 95% confidence intervals. AP, acute pancreatitis; CP, chronic pancreatitis

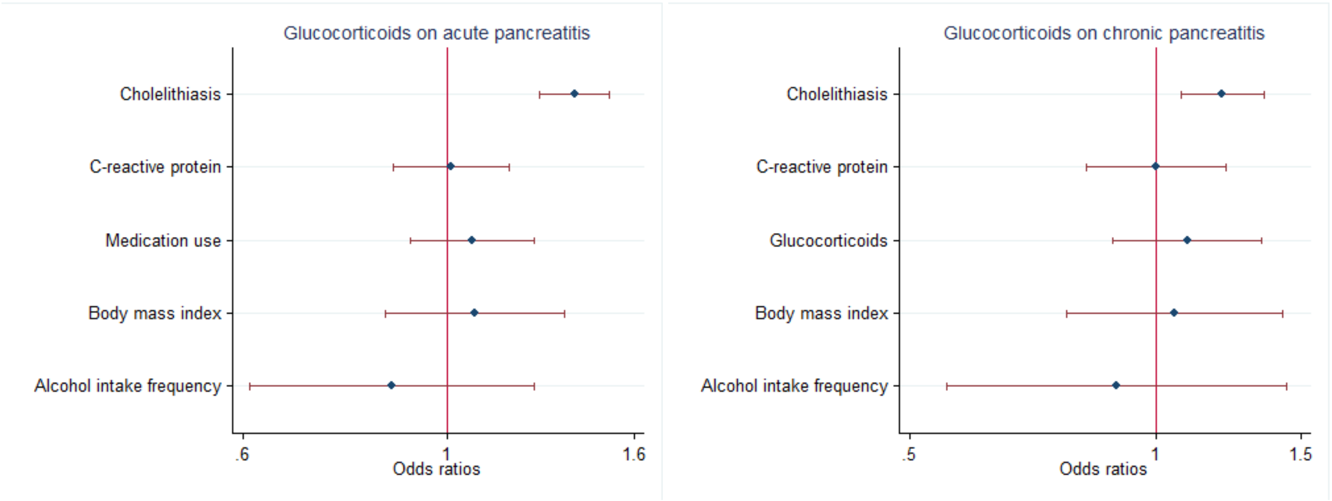

Supplement: Supplementary file 1 [file DataSheet1.pdf]
